# UV-Cured Green Polymers for Biosensorics: Correlation of Operational Parameters of Highly Sensitive Biosensors with Nano-Volumes and Adsorption Properties

**DOI:** 10.3390/ma15196607

**Published:** 2022-09-23

**Authors:** Magdalena Goździuk, Taras Kavetskyy, Daniel Massana Roquero, Oleh Smutok, Mykhailo Gonchar, David P. Královič, Helena Švajdlenková, Ondrej Šauša, Pavol Kalinay, Hamed Nosrati, Migle Lebedevaite, Sigita Grauzeliene, Jolita Ostrauskaite, Arnold Kiv, Bożena Zgardzińska

**Affiliations:** 1 Institute of Physics, Maria Curie-Sklodowska University, 20-031 Lublin, Poland; 2 Department of Biology and Chemistry, Drohobych Ivan Franko State Pedagogical University, 82100 Drohobych, Ukraine; 3 Department of Materials Engineering, The John Paul II Catholic University of Lublin, 20-950 Lublin, Poland; 4 Department of Chemistry and Biomolecular Science, Clarkson University, Potsdam, NY 13699-5810, USA; 5 Department of Analytical Biotechnology, Institute of Cell Biology, National Academy of Sciences of Ukraine, 79005 Lviv, Ukraine; 6 Department of Nuclear Chemistry, Comenius University in Bratislava, 84215 Bratislava, Slovakia; 7 Polymer Institute, Slovak Academy of Sciences, 84541 Bratislava, Slovakia; 8 Institute of Physics, Slovak Academy of Sciences, 84511 Bratislava, Slovakia; 9 Department of Pharmaceutical Biomaterials, School of Pharmacy, Zanjan University of Medical Sciences, Zanjan 45139-56111, Iran; 10 Department of Polymer Chemistry and Technology, Kaunas University of Technology, 50254 Kaunas, Lithuania; 11 Department of Innovation Technologies, South-Ukrainian K.D. Ushynsky National Pedagogical University, 65020 Odesa, Ukraine

**Keywords:** vegetable oil-based polymers, UV curing, positron annihilation, immobilization matrixes, amperometric biosensors

## Abstract

The investigated polymeric matrixes consisted of epoxidized linseed oil (ELO), acrylated epoxidized soybean oil (AESO), trimethylolpropane triglycidyl ether (RD1), vanillin dimethacrylate (VDM), triarylsulfonium hexafluorophosphate salts (PI), and 2,2-dimethoxy-2-phenylacetophenone (DMPA). Linseed oil-based (ELO/PI, ELO/10RD1/PI) and soybean oil-based (AESO/VDM, AESO/VDM/DMPA) polymers were obtained by cationic and radical photopolymerization reactions, respectively. In order to improve the cross-linking density of the resulting polymers, 10 mol.% of RD1 was used as a reactive diluent in the cationic photopolymerization of ELO. In parallel, VDM was used as a plasticizer in AESO radical photopolymerization reactions. Positron annihilation lifetime spectroscopy (PALS) was used to characterize vegetable oil-based UV-cured polymers regarding their structural stability in a wide range of temperatures (120–320 K) and humidity. The polymers were used as laccase immobilization matrixes for the construction of amperometric biosensors. A direct dependence of the main operational parameters of the biosensors and microscopical characteristics of polymer matrixes (mostly on the size of free volumes and water content) was established. The biosensors are intended for the detection of trace water pollution with xenobiotics, carcinogenic substances with a very negative impact on human health. These findings will allow better predictions for novel polymers as immobilization matrixes for biosensing or biotechnology applications.

## 1. Introduction

Currently, the development of bioplastics is one of the fast-growing R&D fields due to the enormous use of plastics worldwide. According to the European Bioplastics Association, in 2019, 2.11 million tons of bioplastics were produced globally, and this number is expected to increase to 2.43 million tons by 2024. The main focus of researchers is the fabrication of bio-based plastics while maintaining their intrinsic biodegradability and low toxicity. Several vegetable oil-based polymeric systems have been developed owing to their inherent biodegradability, low toxicity, and tunability of the polymeric chain [1].

Technogenic pressure leads to the occurrence of one of the most important problems for the environment, which is the contamination of soil, water, and air by toxic chemicals due to pollution of water resources. Especially dangerous products of the chemical and pharmaceutical industry are xenobiotics which are also classified as carcinogens that cause disruption of the endocrine system of humans and animals. They are a matter of industrial origin in the human body, capable of causing effects similar to the effects of high doses of the natural hormone estrogen [2,3]. Mimicking estrogen, xenobiotics adversely affect the function of the endocrine system and are able to cause various health defects, affecting synthesis, metabolism, and cellular reactions of natural estrogens [4,5,6,7].

As an example, xenobiotic Bisphenol A is used for the manufacture of polycarbonate plastic and epoxy resins, which are raw materials for the production of packaging materials for food and drinks. The world market for Bisphenol A is over 6.4 billion pounds per year, and thus, it is one of the chemicals with the highest volume of production all over the world [8]. Bisphenol A is released into the environment, including wastewater and seawater, resulting in a widespread negative impact on humans and animals. The development of new approaches for monitoring these dangerous substances coming from wastewater is a topical problem to improve human life first of all.

The use of vegetable oils as starting materials for the preparation of polymers is widely spread due to their inherent biodegradability, low toxicity, and ability to modify functional groups [9]. Curing reactions of raw oils take a long time due to their low reactivity. However, the curing time can be shortened by using oils with more reactive functional groups, for example, epoxidized oils [9]. Vegetable oil-based photopolymers could be used as a holding matrix in biosensors. Photopolymerization shortens the reaction time from hours to minutes compared to thermal polymerization [10]. The photocurable formulation consists of a monomer, a reactive diluent, which reduces the viscosity and participates in the reaction, and a photoinitiator, which absorbs the light and generates the reactive species [11,12]. The use of photopolymerization has a number of advantages for the process of modern materials’ production, e.g., energy saving, fewer side reactions, short time, easy setup, lower cost, etc. [13,14].

It has been recently shown [15] that vegetable oil-based photopolymers can be successfully used as an immobilization matrix for biosensing applications. The nanostructure of polymer matrixes used for sensor construction plays a key role in obtaining biosensors with high sensitivity and selectivity [15,16,17,18]. Therefore, a deep understanding of the polymer nanostructure properties will make the selection of optimal materials for the intended applications in the construction of biosensors simpler.

Nanostructure studies of polymer matrixes dedicated to the production of biosensors were carried out using Positron Annihilation Lifetime Spectroscopy (PALS). This technique is used to study the microstructure of polymers [19]. The simplicity and non-destructiveness of the analysis have increased the interest in using PALS in polymer science. The technique examines positron annihilation in the material of interest, measuring the lifetimes of positrons or their bounded state with an electron, so-called positronium (Ps). Positronium is formed in most polymers, and, in particular, the triplet state Ps; o-Ps is an important probe for the investigation of the local space between the polymer chains [20,21]. The experimentally obtained lifetime of o-Ps (*τ_o-Ps_*) enables the determination of the average size of local free volumes [22] (even closed) in the range of 0.1–50 nm. In addition, the size distribution and concentration can be estimated using suitable models [23,24,25,26,27,28,29,30,31,32]. The size of free volume in the medium (assuming spherical geometry) can be estimated based on the Tao–Eldrup equation [23,24,25,26]:

(1) $$\tau_{3}=\frac{1}{\lambda_{b}}{\left[1-\frac{R}{R+\Delta }+\frac{\sin\left(2\pi R/R+\Delta \right)}{2\pi }\right]}^{-1},$$

where *λ_b_* = 2 ns^−1^ is the decay constant in bulk, *R*-the radius of free voids, and Δ = 0.166 nm is an empirical constant [24]. The volume of free spaces in the medium:

(2) $$V=\frac{4}{3}\pi R^{3},$$

allows to calculate the relative free volume fraction, *f_V_*:

(3) $$f_{V}=CI_{3}V,$$

where *I_3_* is the o-Ps intensity, and *C* is a parameter that differs in the range of 0.001–0.002 depending on the nature of the material [33,34]. For example, we found that polystyrene has *C* = 0.0014 by using a similar calibration method [34].

The temperature dependence of the annihilation parameters can be used to obtain additional information on the material properties, such as transition temperatures (phase and dynamic transitions). Combining PALS with other techniques, e.g., dilatometry [35], the shape of the pores or the anisotropy of their extensibility can also be obtained [36].

The free volume properties of different matrixes, suitable for the construction of enzyme biosensors based on laccase, were studied by PALS recently [15,16,17,18,37,38]. Positron annihilation has also been used in this work to characterize selected matrixes, their stability, exposure to temperature, and the influence of humidity. The obtained results regarding the local free volume and adsorption properties of vegetable oil-based photopolymers should be taken into account for further construction of amperometric enzyme biosensors using these materials.

In the present study, we report the results of a detailed characterization of two polymeric systems obtained by cationic and radical photopolymerization reactions: linseed oil-based (ELO/PI, ELO/10RD1/PI) and soybean oil-based (AESO/VDM, AESO/VDM/DMPA). The polymers were tested as immobilization matrixes in the construction of laccase-based amperometric biosensors. A direct dependence of the biosensor’s main operational parameters and the polymer microstructural characteristics were demonstrated. This finding confirms the possibility of controlling the functionality of amperometric enzyme biosensors using the microstructure characterization of an immobilization polymer matrix.

## 2. Experimental

### 2.1. Materials

Epoxidized linseed oil (ELO, having an average number of 6 epoxy groups per molecule) was purchased from Chemical Point. Acrylated epoxidized soybean oil (AESO, an average number of acryloyl groups per molecule calculated from ^1^HNMR spectrum is 2.7 and 0.3 of epoxy groups), trimethylolpropane triglycidyl ether (RD1), triarylsulfonium hexafluorophosphate salts (PI, mixed, 50% in propylene carbonate), 2,2-dimethoxy-2-phenylacetophenone (DMPA), laccase from *Trametes versicolor* with the activity of 13 U·mg^−1^, and 2,2′-azino-bis(3-ethylbenzthiazoline-6-sulfonic acid) (ABTS) were purchased from Sigma-Aldrich. Vanillin dimethacrylate (VDM) was purchased from Specific Polymers. Tetrahydrofuran (THF) (99.9%) was purchased from Eurochemicals. All materials were used without further purification. 

### 2.2. Preparation of Samples

**Figure 1:**
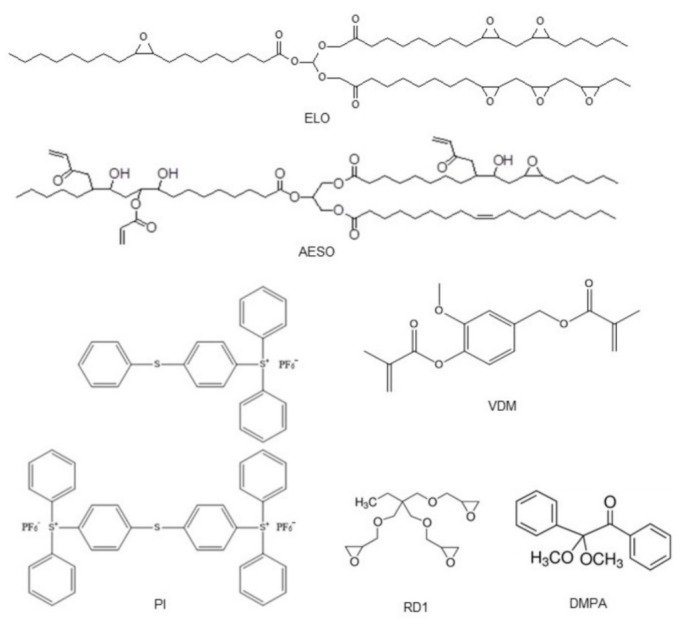
Chemical structures of epoxidized linseed oil (ELO), acrylated epoxidized soybean oil (AESO), trimethylolpropane triglycidyl ether (RD1), vanillin dimethacrylate (VDM), triarylsulfonium hexafluorophosphate salts (PI), and 2,2-dimethoxy-2-phenylacetophenone (DMPA).

Linseed oil-based (ELO/PI, ELO/10RD1/PI) and soybean oil-based (AESO/VDM, AESO/VDM/DMPA) polymers were obtained by cationic and radical photopolymerization reactions according to the previously reported procedures [39,40,41]. Irradiation in a wavelength range of 250–450 nm was used with an intensity of 310 mW·cm^−2^. In order to improve the cross-linking density of resulting polymers, 10 mol.% of trimethylolpropane triglycidyl ether (RD1) was used as a reactive diluent in cationic photopolymerization of ELO. Vanillin dimethacrylate was used as a plasticizer in radical photopolymerization reactions of AESO (molar ratio of monomers 1:0.5). In this study, 3 mol.% of PI as a photoinitiator was used in the preparation of linseed oil-based polymers. Polymer AESO/VDM was prepared without a PI photoinitiator, and 3 mol.% of DMPA were used in the preparation of polymer AESO/VDM/DMPA. The use of photoinitiator DMPA led to obtaining more cross-linked polymers. The cross-linking density of polymers is improved by the photoinitiator concentration increasing to a certain extent, then decreasing due to a primary radical’s termination reaction [42]. The 3 mol.% was an optimal concentration of DMPA to initiate the photopolymerization of acrylates [43]. Chemical structures of ELO, AESO, RD1, VDM, PI, and DMPA are presented in Figure 1. The obtained polymer samples with a diameter of 15 mm and a height of 3 mm were transparent and had a yellowish color (Figure S1, supplementary material). 

### 2.3. PALS Technique

The spectrometer equipped with two Hamamatsu scintillation heads with BaF_2_ crystals set at an angle of 90° was used for the Positron Annihilation Lifetime Spectroscopy (PALS) measurements. The resolution of the system was 240 ps. Spectra collected every 3/24 h had statistics 7–8 × 10^6^ per spectrum. The ^22^Na positron source was enclosed in 7 μm thick Kapton® film. The source activity was 0.6 MBq. Biopolymers and the source (in “sandwich” geometry) were placed in the measuring chamber (Figure S2, supplementary material). The chamber allowed making measurements in a broad range of temperatures—here from −150 °C to +40 °C under atmospheric pressure, vacuum, water vapor dosing, or in the presence of liquid water. The temperature was stabilized with an accuracy of 0.1 K by the Shimaden FP21 controller. The typical measuring cycle began at room temperature (RT) and atmospheric pressure. Afterward, the temperature was decreased abruptly (5 K per minute) to −150 °C. At this temperature, the stability of the sample was checked (for 24 h). Then the temperature was increased stepwise every 10 °C to +40 °C and flowingly lowered to −150 °C in the same step. After a sudden increase in temperature again to room temperature, measurements were carried out first to check if the temperature-induced changes had occurred in the sample. The next measurements were carried out as: dosing vapor water, soaking a sample with liquid water, and at the end in a vacuum. In the sorption experiment performed, double-deionized water (18 MΩcm) was adsorbed into the samples. Water dispensed to the sample was previously devoid of paramagnetic oxygen molecules in the process of degassing the sample by the freeze-thaw technique (Figure S3, supplementary material).

The spectra were analyzed using the LT 9.2 [44] (all spectra) and the MELT [45,46] software (only the spectra with high statistics—more than 5 × 10^7^ counts—were used for maximum entropy lifetime analysis MELT). In the LT software, two ways of analysis were tested, assuming the presence of three well distinguishable components: the shortest one attributable to the para-positronium (with the p-Ps lifetime *τ*_1_ and intensity *I*_1_), the intermediate one associated with free positron annihilation (with the e^+^ lifetime *τ*_2_ and intensity *I*_2_), and the longest one exceeding 1 ns assigned to the ortho-positronium (with the o-Ps lifetime *τ*_3_ and intensity *I*_3_). In the first attempt, it was assumed that all components remained free; in the second attempt, the ratio of *I_p_*_-Ps_: *I_o_*_-Ps_ = 1:3 was adopted.

### 2.4. Swelling

A swelling experiment was done with pure water (CHROMASOLV™, LC-MS Ultra from Honeywell/Riedel-de Haen). Samples were weighted as received at room temperature (23 °C), then were immersed into water (in Petri dishes) and periodically, with a step of several hours, were wiped off the surface water, weighed, and re-immersed in water. This was repeated until a saturated state (or approximately saturated state) was reached. The time of weight measurements was recorded. Semimicro balance SMG 425i (VWR) was used for weighing during the swelling experiment.

### 2.5. PALS Spectrometer for Desorption Experiments after Swelling

The PALS spectrometer for desorption experiments after swelling consisted of detectors based on BC-422Q Saint-Gobain Crystals plastic scintillators, Scionix XP2020UR photomultipliers, and Ortec electronics. The time resolution of the spectrometer (320 ps FWHM) and the correction for annihilation in the positron source (1 MBq, Kapton^®^ film, thickness of 8 μm) was made using a defect-free Al standard with a single lifetime of 166 ps. The sample was hermetically sealed in a sandwich arrangement in an Al chamber to prevent water from escaping from the sample during the measurement. Time spectra were evaluated using the LT program. A complex component was used, fixing the ratio of intensities for p-Ps and o-Ps in the ratio 1:3.

### 2.6. Biosensors Preparation and Evaluation

Amperometric biosensors were evaluated using constant-potential amperometry in a three-electrode configuration with an Ag/AgCl/KCl (3 M) reference electrode and a Pt-wire counter electrode. Amperometric measurements were carried out using a potentiostat CHI 1200A (IJ Cambria Scientific, Burry Port, UK) connected to a personal computer and performed in a batch mode under continuous stirring in a standard 40 mL electrochemical cell at room temperature. Graphite rods (type RW001, 3.05 mm diameter, area 7.3 mm^2^, Ringsdorff Werke, Bonn, Germany) were used as working electrodes. Before sensor preparation, the graphite electrodes were polished with emery paper and cleaned with distilled water.

The laccase immobilization was done as follows: dropping 5 µL of laccase solution (1 mg·mL^−1^ with an activity of 13 U·mg^-1^) on the working electrode surface and drying for 5 min at 15 °C; covering the dried enzyme by 0.3 mm thick layers of photocross-linked polymers ELO/PI, or ELO/10RD1/PI and fixation of the formed enzyme–polymer matrix by net cap using plastic “O”-ring [15]. The glue-like AESO/VDM/DMPA was smeared on the enzyme-formed layer without any additional immobilization implements. The formed bioelectrodes were washed by 50 mM acetic buffer, pH 4.5, and stored at 8 °C till use.

## 3. Results and Discussion

### 3.1. Investigation of Sample Stability

**Figure 2:**
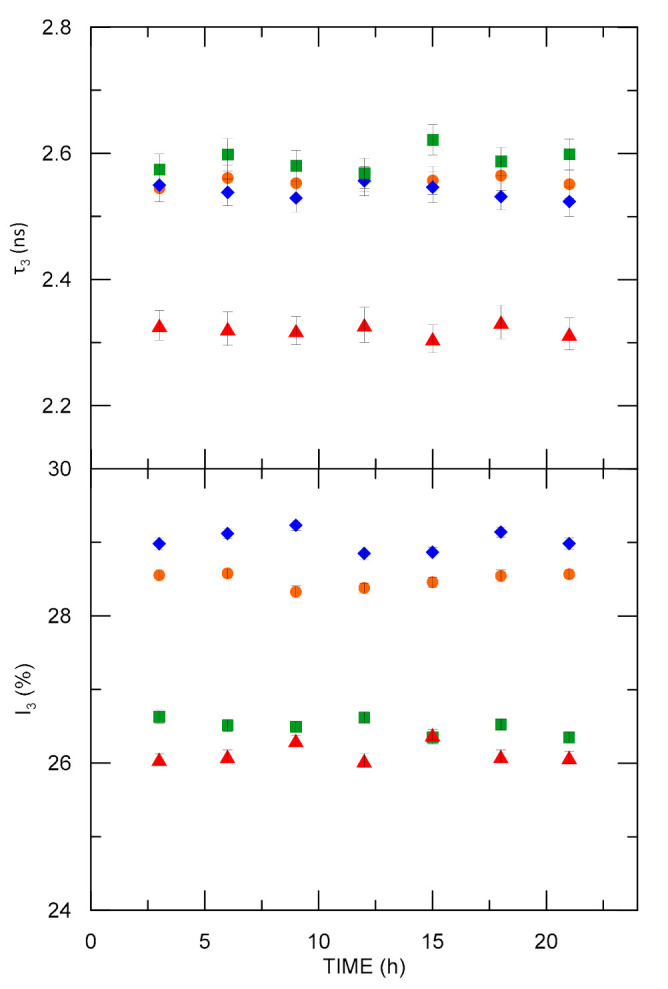
The o-Ps lifetime and intensity as a function of time for the four investigated samples at 18 °C; dots–ELO/PI, diamonds–ELO/10RD1/PI, squares–AESO/VDM, and triangles—AESO/VDM/DMPA.

The PALS parameters of all four samples were tested at 18 °C as a function of time to verify polymer matrixes’ stability. As can be observed in Figure 2, all the tested samples showed great stability. Similar sizes of free volumes were obtained for the ELO/PI, ELO/10RD1/PI, and AESO/VDM samples. The samples based on epoxidized linseed oil showed higher o-Ps intensity. Over time, the PALS parameters change for each sample and do not go beyond the measurement error. Therefore, the spectra measured for each of the samples at 18 °C were summed up to obtain spectra with greater statistics and then analyzed by the LT and MELT software.

From a temperature of 18 °C, the samples were cooled down to −150 °C, and a stability test at this temperature was performed. A number of materials (including polymers) show the instability of PALS parameters as a function of exposure time to irradiation, which is explained by the low-temperature electron trapping effect [47,48,49,50,51]. This effect is manifested by an increase in the intensity of the o-Ps component as a function of time. This effect was not observed in any of the investigated matrixes demonstrating high stability at low temperatures (see Figure S4, supplementary material). 

The effect of exposure of samples to drastic changes in temperature was investigated. As mentioned above, low temperatures did not affect the stability of the systems. The nanostructure of the virgin sample and the stress sample (subjected to the process of cooling down to −150 °C and then reheated to +40 °C and then to RT) remained unaltered, as reflected in the lifetime and intensity of o-Ps. The spectra for the virgin sample, samples at −150 °C, and samples reheated to RT were summed up and analyzed by the LT and MELT software.

**Figure 3:**
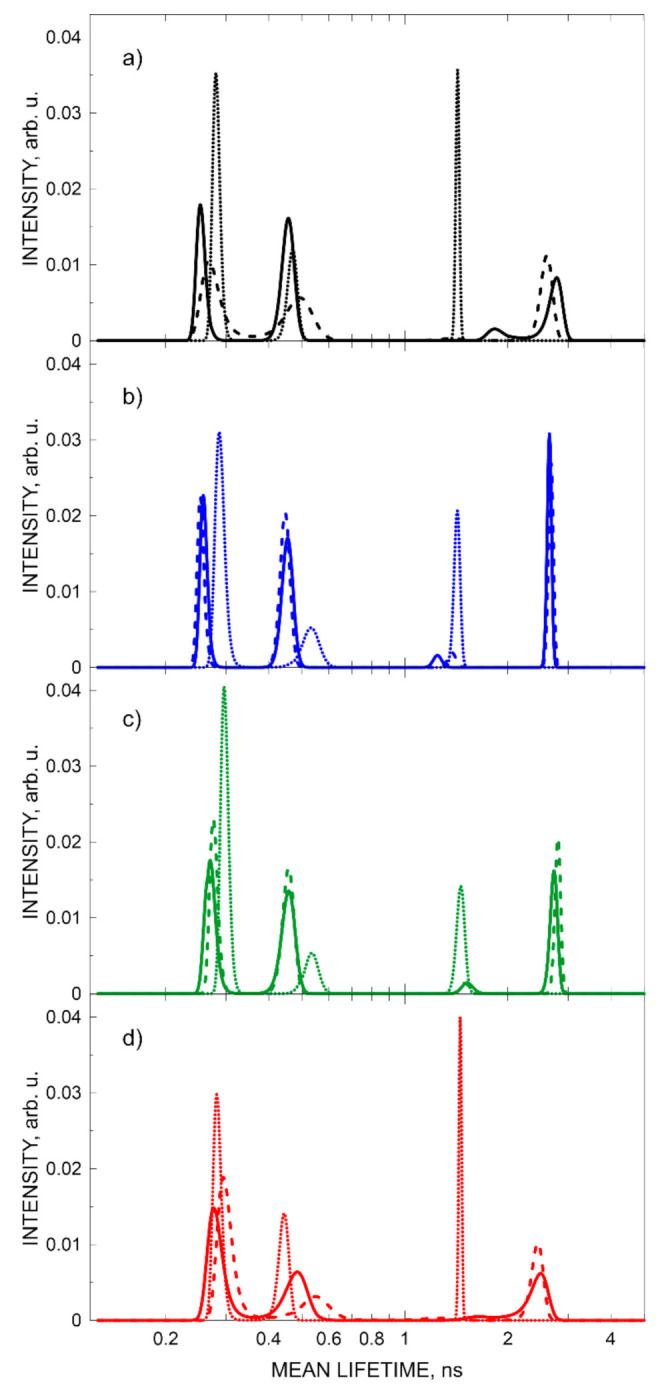
Distribution of mean lifetimes in the PALS spectrum of samples (**a**) ELO/PI, (**b**) ELO/10RD1/PI, (**c**) AESO/VDM, and (**d**) AESO/VDM/DMPA obtained from MELT processing of the spectra. Virgin sample at 18 °C (solid line), sample at −150 °C (dotted line), and again at room temperature (dashed line).

The distribution of the mean lifetimes in the PAL spectrum of each sample at selected temperatures, obtained from MELT processing of the spectra, are shown in Figure 3. There are no PALS parameter changes between the virgin sample and the sample subjected to the thermal treatment process (solid and dashed lines in Figure 3, respectively). The MELT analysis showed the presence of four components (two o-Ps components) at RT in three out of four samples. The third component has a very low intensity, between 0.8 and 4.2%, and a lifetime exceeding 1 ns. The results obtained for the last tested sample (AESO/VDM/DMPA) suggest the presence of only three PALS components—one o-Ps component. In this sample, o-Ps lifetime is remarkably shorter compared with the other samples. At a lower temperature (Figure 3 shows results at −150 °C), there were only 3 components in PALS spectra, with the o-Ps component shortening in each case to approximately 1.4 ns (dotted lines in Figure 3). 

The MELT analysis showed the presence of an additional short-lived o-Ps component in the spectra at RT. However, the very low intensity and high variability of this component (lifetime of o-Ps varied from 1.2 to 1.9 ns) prevented its identification in spectra with approx. 6–12 times lower statistics. The challenge of distinguishing the low-intensity o-Ps component together with its disappearance in the low-temperature phase and its clear variability prompted us to adopt spectral analysis by LT software with a three-component distribution.

It is worth studying the obtained values of short-lived components that showed longer than expected. The first of the PALS components is usually attributed to p-Ps. Its high value is due to the summation effect of the p-Ps component (0.125 ns) and the free annihilation or component associated with e−/e+ trapping. In addition, the high intensity of this component confirms that the theoretically expected ratio *I_p_*_-Ps_:*I_o_*_-Ps_ = 1:3 is not maintained. 

Theoretically, the ratio *I*_1_:*I*_3_ = 1:3 would be expected. However, MELT analysis showed that this ratio is not preserved. In fact, the ratio *I_1_*: *I_3_* ranges from 1.51 to 3.04. Comparing the spectrum analysis using LT (assuming *I*_1_:*I*_3_ = 1:3) and MELT (free components) software, the o-Ps parameters resulted in being similar. In the LT 9.2 analysis with distribution into three components and assuming *I*_1_:*I*_3_ = 1:3, the *I*_3_ was overestimated by 1.23% and 2.82% at RT and −150 °C, respectively. Additionally, o-Ps lifetimes were reduced by 84 ps. Nevertheless, this analysis improved the values of short-lived components, $\tau_{1}{}_{1:3}=0.160\;\mathrm{ns}\;$and $\tau_{2}{}_{1:3}=0.369\;\mathrm{ns}$, compared to $\tau_{1}{}_{\mathrm{free}}=0.287\;\mathrm{ns}$ and $\tau_{2}{}_{\mathrm{free}}=0.541\;\mathrm{ns}$. Not maintaining the ratio *I*_1_:*I*_3_ = 1:3 significantly affects the parameters of the two short-lived components without noticeable modification of the o-Ps parameters. Therefore, the adoption of the above-mentioned assumption did not affect the determined PALS parameters of the o-Ps component but actually improved the spectrum matching parameters and reduced the experimental data dispersions.

In summary, comparing the results of distribution into three components with and without the expected *I*_1_:*I*_3_ ratio, we decided to move forward assuming *I*_1_:*I*_3_ = 1:3 in the analysis of PALS spectra.

### 3.2. Temperature Influence Analysis

**Figure 4:**
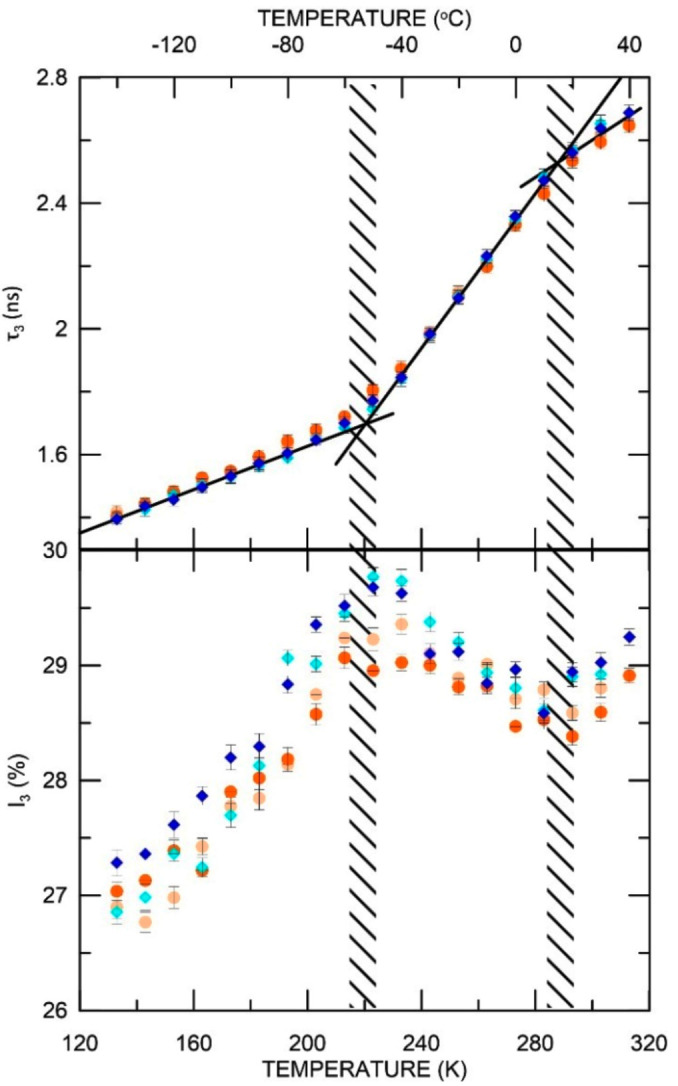
The o-Ps lifetime and intensity as a function of increasing (ELO/PI—orange dots, ELO/10RD1/PI—dark blue diamonds) and decreasing (ELO/PI—creamy dots, ELO/10RD1/PI—light blue diamonds) temperature.

After the temperature jumped from 18 °C to −150 °C, in which the sample showed stability of PALS parameters for 24 h (Figure S4, supplementary materials), a cycle of increasing and decreasing temperature in the range −150 °C and +40 °C was carried out. As seen in Figure S5 (supplementary material), in this range of temperature, the first phase transition (glass transition, *T*_g_) is visible in ELO/PI at ~220 K (−53 °C) and the second one at temperature 293 K (20 °C) which is marked on the figure along with o-Ps intensity. The hysteresis effect in the sample is absent or negligible. The values of the estimated *T_g_* for all samples as a function of increasing and decreasing temperature are gathered in Table S1 (Supplementary materials). An analogous cycle of temperature measurements was made for the ELO/10RD1/PI sample. As shown in Figure S6 (supplementary materials), the first phase transition (glass transition, *T*_g_) is visible at 220 K (−53 °C) and the second one at 283 K (10 °C). Figure 4 illustrates both ELO/PI and ELO/10RD1/PI temperature-dependent measurements. The phase transition temperatures determined by the PALS technique were compared with the results of the DSC analyzes (Figure S7, supplementary material).

The matrix modifications (ELO/10RD1/PI sample) do not affect the matrix nanostructure since changes in the o-Ps lifetime as a function of temperature in both samples are very similar. However, slight divergences can be observed in *I_3_(T)* function. Structural modification of the matrix (changing the chemical composition) increases the value of o-Ps intensity. This could imply either an increase in the number of free volumes or an increase in the chances of creating a positronium atom. This may be an onset of *τ*_3_ saturation regime, which is often observed above the glass transition temperature. In this regime, the lifetime of o-Ps becomes comparable with the segmental relaxation time, which controls the dynamics of the holes. This limits the use of the PALS technique in the rubbery or molten state of polymer samples.

**Figure 5:**
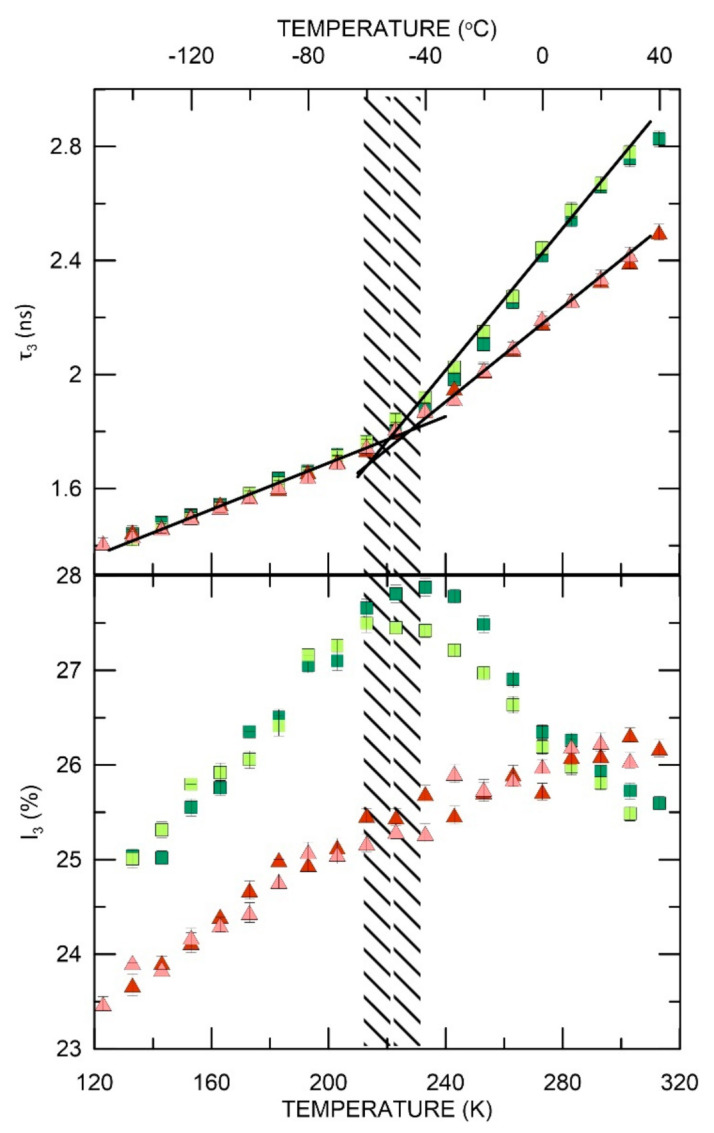
AESO/VDM, AESO/VDM/DMPA: the o-Ps lifetime and intensity as a function of increasing (AESO/VDM—dark green squares, AESO/VDM/DMPA—red triangles) and decreasing (AESO/VDM—light green squares, AESO/VDM/DMPA—pink triangles) temperature.

The AESO/VDM sample showed PALS parameters stability after the rapid change in temperature from 18 °C to −150 °C in 24 h (see Figure 2 and Figure S4, supplementary material). Then, the PALS parameters were tested during the cycle of increase and decrease of temperature in the range of −150 °C and +40 °C. As shown in Figures S8 and S9 (Supplementary material), the phase transition (*T*_g_) is located at 223 K (−50 °C). We found divergences in o-Ps intensity between results obtained while increasing and decreasing the temperature between 223 K and 263 K (−10 °C). Nevertheless, in both cases, there was a reduction of o-Ps intensity above 233 K (−40 °C). In samples AESO/VDM/DMPA and AESO/VDM, no difference in lifetime values during the temperature measurement cycle were observed. The *T_g_* (indicated in Table S1, supplementary material) is well visible in PALS analysis, and the next phase transition occurs at higher temperatures (for AESO/VDM/DMPA, it is above the range of temperatures analyzed by the PALS technique, see Figure 5). There is also a higher o-Ps intensity in sample AESO/VDM than in AESO/VDM/DMPA below 273 K (0 °C).

In Figure S10 (Supplementary material), all temperature-dependent PALS parameters for the tested samples are shown. The highest values of o-Ps intensity can be observed for the ELO/10RD1/PI sample and the lowest ones for AESO/VDM/DMPA sample, but they become bigger than AESO/VDM o-Ps intensities above 273 K (0 °C). The o-Ps parameters were used to calculate, using Equation 2 and Equation 3, free volume sizes *V* and the relative free volume fraction, *f_V_*, respectively.

**Figure 6:**
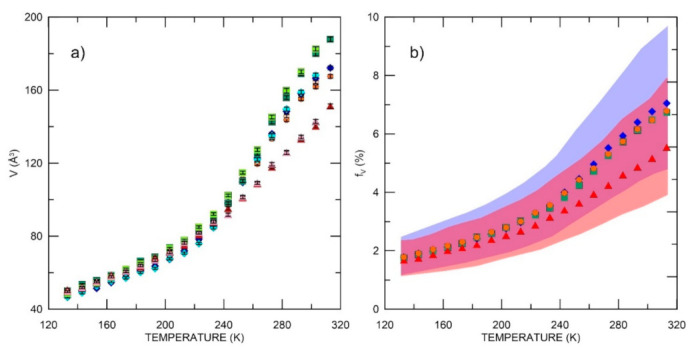
Free volume (**a**) and relative free volume fraction (**b**) as a function of temperature in increasing temperature: ELO/PI—orange dots, ELO/10RD1/PI—dark blue, diamonds, AESO/VDM—dark green squares, AESO/VDM/DMPA—red triangles; and decreasing of temperature: ELO/PI—creamy dots, ELO/10RD1/PI—light blue diamonds, AESO/VDM—light green squares, AESO/VDM/DMPA—pink triangles. In the figure to the right, the points correspond to *C* = 0.0014. For *C* in the range 0.001 to 0.002, the blue and red areas marked results for ELO/PI and AESO/VDM/DMPA, respectively.

The results (Figure 6) show that the volume of free spaces (determined on the basis of Equations 1 and 2) at low temperatures is similar in all investigated samples. However, above *T_g_*, the largest increase in size is observed for ELO-based samples. In AESO-based samples at higher temperatures than *T_g_*, the discrepancy in free volume sizes is remarkable. The polymer matrix (AESO/VDM) clearly showed larger free volumes, even compared to samples based on ELO. Chemical modification of AESO/VDM (sample AESO/VDM/DMPA) led to a decrease in free volumes. The DMPA photoinitiator contains aromatic nuclei, which, when incorporated into the network at higher temperatures than the *T*_g_, can interact stronger with the environment and effectively reduce the free volumes [52]. On the other hand, a more pronounced decrease of *I*_3_ in the whole range and a different temperature dependence of *I*_3_(*T*) in comparison with other samples can be explained by the influence of chemical changes along the polymer chains. This leads to the suppression of Ps formation due to the presence of inhibitors (oxygen atoms from the reacted DMPA) which cannot be associated with a decrease in free volume numbers.

The relative free volume fraction (*f_V_*) increases at a similar rate for all samples except AESO/VDM/DMPA (see discussion above). The points correspond to *f_V_* when *C* = 0.0014, but the marked areas (blue and red field in Figure 6) correspond to *C* = 0.001 and 0.002 for ELO/PI and AESO/VDM/DMPA, respectively. As can be seen, of the four tested samples, only AESO/VDM/DMPA clearly shows a smaller size of free volumes and lower relative free volume fraction.

### 3.3. Temperature Influence Analysis

The sorption/desorption process was followed in order to test the influence of water in the polymeric samples:

1. At 18 °C, the samples were placed in H_2_O vapor, and the process of water sorption from vapor was examined;
2. After 2–3 days, deionized water was poured into the chamber, and the evolution of the sample nanostructure over time was investigated;
3. The last step was water removal from the sample chamber and vacuuming out the remaining (bound in the sample) water.

**Figure 7:**
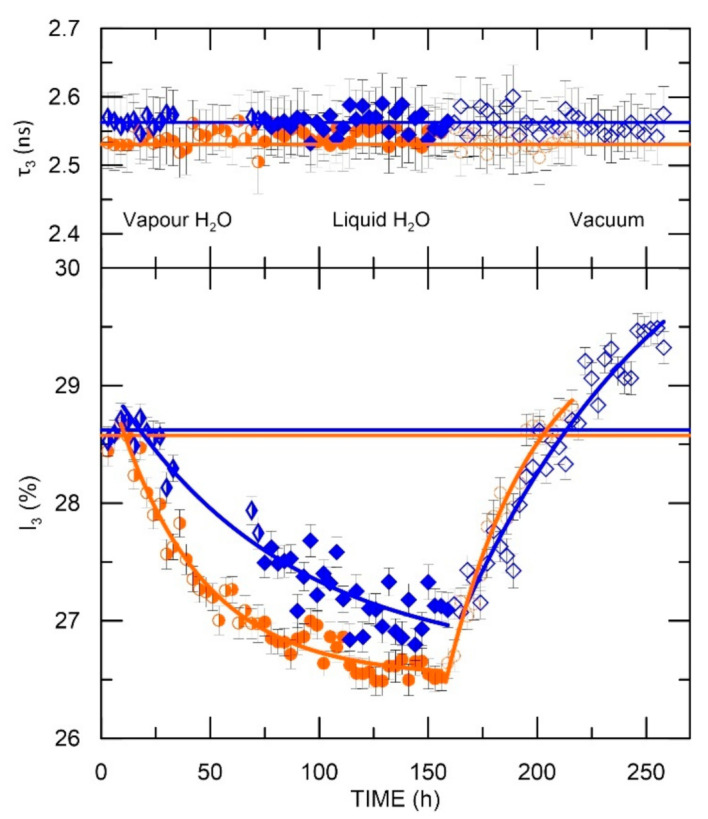
Dependence of o-Ps lifetime and intensity on water presence (ELO/PI—dots, ELO/10RD1/PI—diamonds); half-full points, full points, and empty points represents stage 1, 2, and 3 of measurement regime, respectively.

**Figure 8:**
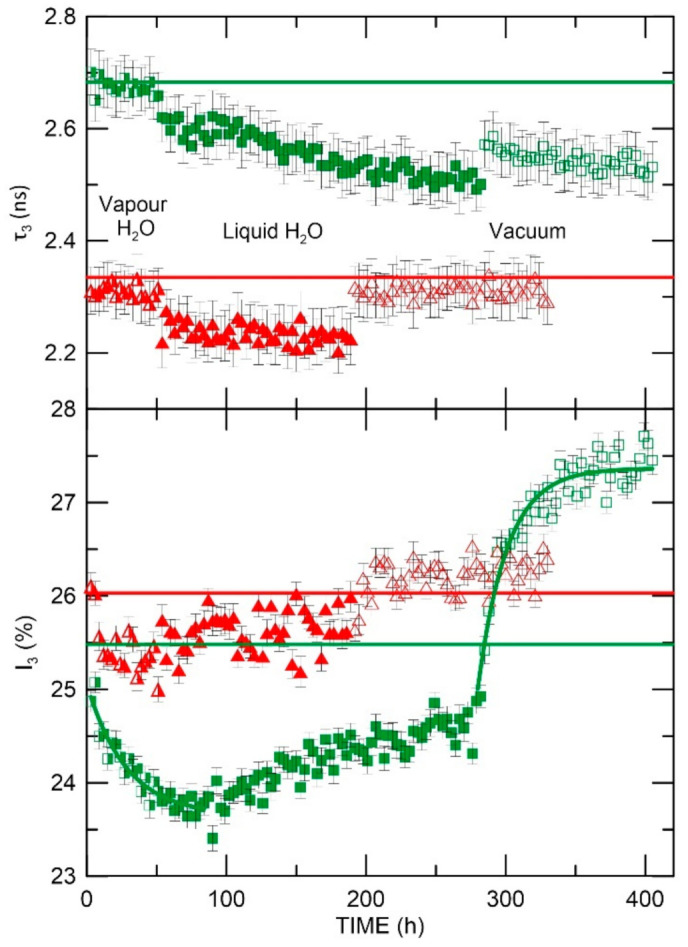
Dependence of o-Ps lifetime and intensity on water presence (AESO/VDM—squares, AESO/VDM/DMPA—triangles); half-full points, full points, and empty points represents the stage 1, 2, and 3 of measurement regime, respectively.

The water sorption/desorption process in the samples is shown in Figure 7 and Figure 8. The horizontal lines are the reference values of PALS parameters for samples at 18 °C before conducting the above-mentioned process. Compliance of these reference levels with the initial PALS parameter values in Figure 7 and Figure 8 (first points) confirm that the temperature has no destructive effect on the samples. The reference level allows as well analysis of the impact of the presence of water molecules on the sample nanostructure. Figure 7 and Figure 8 display three different areas that are correlated with the procedure described above. It is worth mentioning that water molecules are paramagnetic, i.e., the ortho-para conversion process is inevitable. However, the conversion effect was not introduced by oxygen molecules from the air dissolved in the water—the freeze-thawing was used to remove oxygen from the environment and avoid its effects in the measurements.

For ELO-based samples (Figure 7), we observed o-Ps lifetime stability during the sorption/desorption process. The presence of H_2_O molecules does not change the size of the free volumes in these polymers. However, it is a key factor in altering the o-Ps intensity. In the process of sorption of water from vapor (stage 1), a decrease in the intensity by 2% is observed in both ELO/PI and ELO/10RD1/PI samples (Figure 7, half-full filled dots and diamonds, respectively). Flooding the sample with water (stage 2, full field points in Figure 7) did not speed up the process, as evidenced by slight changes in the intensity of o-Ps at the second stage of the measurement regime. This could imply that the process of water sorption by the polymer from vapor and liquid H_2_O follows the same mechanism, i.e., migration of water molecules through/into the polymer structure. In the ELO/10RD1/PI sample, the intensity decrease is less pronounced. This can be explained by the higher hydrophobicity of the polymer (CH_3_ groups along the chain) that hinders water diffusion.

Physical removal of water from the sample environment, followed by the removal of water molecules from the sample in the degassing process (desorption, stage 3), led to an increase over time of the intensity. 

An exponential fitting of the curve perfectly matches with the process of water sorption (desorption) in both ELO-based samples:

(4) $$I_{3}=I_{30}+I_{3f}\left(1+\exp\left(-\frac{t}{\theta }\right)\right),$$

where $I_{30}$ is the value at which *I*_3_ strives; $I_{3f}$ is the maximum decrease (increase) in o-Ps intensity observed as a result of the sorption (desorption) process; the sum $I_{30}+I_{3f}$ represents the initial o-Ps intensity (reference level), and *θ* is time constant of the sorption (desorption) process (Table 1).

As it can be observed, the time constant of the sorption and desorption process is similar in the ELO/PI sample (≈ 40 h), while in the ELO/10RD1/PI sample, the desorption process is extended in time compared to the sorption process. In both samples, during the sorption process, the o-Ps intensity decreases by 2.50% (±0.02). The desorption process was carried out for 72–80 h. Therefore, it can be assumed that the I_3_ values achieved are not saturation values, but there was no scientific interest in prolonging the measurement for longer times.

As Figure 8 shows, the AESO-based polymers displayed a more complex change in PALS parameters. The evolution of both o-Ps lifetimes and intensities was analyzed. Both samples in the presence of H_2_O vapor (stage 1) showed high stability of o-Ps lifetime and a decrease in intensity (smaller than in the case of ELO-based samples). This indicates a change in the polymer structure that impairs the diffusion of water into the free volumes of the polymer. Despite the large free volume at RT in AESO/VDM and the comparable *f_V_* fraction to ELO-based samples, the more hydrophobic network structure of AESO-based samples in comparison with ELO-based samples caused low water sorption.

After flooding, the AESO/VDM sample (stage 2) showed first the stepwise changes of *τ*_3_ and then a linear relationship of o-Ps lifetime changes as well as intensity. The shortening of o-Ps lifetime by about 100 ps during 180 h of measurement was as well displayed together with a linear increase in the intensity of approximately 1%. The different changes observed in the presence of water vapor and liquid H_2_O suggest a different process/mechanism of water sorption to the sample. The determined time constant of the sorption process was 28 h (Table 1), which was a much lower value than in the case of ELO-based polymers.

In the AESO/VDM sample, a significant change in o-Ps intensity and lifetime can be noted in the desorption process (stage 3). The intensity increases exponentially with a time constant of 21 h and stabilizes at 27.4% (significantly higher than the reference level). The o-Ps lifetime increases stepwise but does not reach the values observed at the beginning of the measurement cycle. This could indicate that water has been bound to the sample and has permanently modified the sample nanostructure leading to a reduction in the size of free volumes. Significant changes in o-Ps intensity values may be an indicator as well of the removal of chemically unbound water from the matrix.

The rapid sorption and leakage of water, as well as a smaller amount of absorbed water, can also be explained by the fact that the water is mostly localized in surface cavities. The final value of *I*_3_, which is higher than the initial one, may also be due to the release of unreacted and volatile/evaporable parts from the volume of the network during the penetration of water into the polymer network. A gradual increase after 100 h (a decrease or saturation of *I_3_* in stage 2 would be expected) can be explained by the gradual dilution of the water by unreacted parts in the matrix after a certain time of contact of the sample with liquid water.

During desorption in a high vacuum, not only water but also other volatile substances are removed, and therefore *I*_3_ in the final state is not identical to the initial one. The slight decrease of *τ_3_* during desorption, as well as the decrease of *τ*_3_ in the final state compared to the initial value, indicates that this is not simply the desorption of water but that the properties of the matrix have been modified.

The AESO/VDM/DMPA sample after flooding (stage 2) displayed high stability of both PALS parameters (*τ_3_* and *I_3_*), with a visible change (stepwise shortening) of o-Ps lifetime. A stepwise change in o-Ps lifetime and intensity occurs when water is removed from the sample environment (stage 3). The o-Ps parameters in the sample returned to their preliminary level (before sorption measurement) and remained stable. This nature of the changes suggests that water is not absorbed into the sample. In addition, the observed effect of shortening o-Ps lifetime and intensity in the presence of water molecules can be interpreted by positron annihilation in water. Then the measured parameters of PAL spectra give averaged information about two types of free volumes in which o-Ps annihilates. The first belongs to the polymer matrix and the second one to bubbles produced in water by positronium. All these analyses suggest that there is no water sorption in this sample. This finding would enable this material to be discarded for the construction of biosensors.

The process of water penetration as well as its release (together with some unreacted portion from the matrix), as shown in Figure 8, (initial and final value of *I_3_*) is significantly blocked in AESO/VDM/DMPA due to the smallest free volume at RT.

### 3.4. Swelling Process and Subsequent Water Desorption for PALS Analysis

The objectives of the swelling studies were (i) to determine the amount of water that the sample is able to absorb from the liquid environment; (ii) to determine the time course of water sorption; (iii) to estimate the diffusion coefficients of water in the examined samples and (iv) to qualitatively analyze their crosslinking density. PALS measurements (the process of desorption) after swelling can reveal the behavior of the free volume properties of the polymers with different water content.

**Figure 9:**
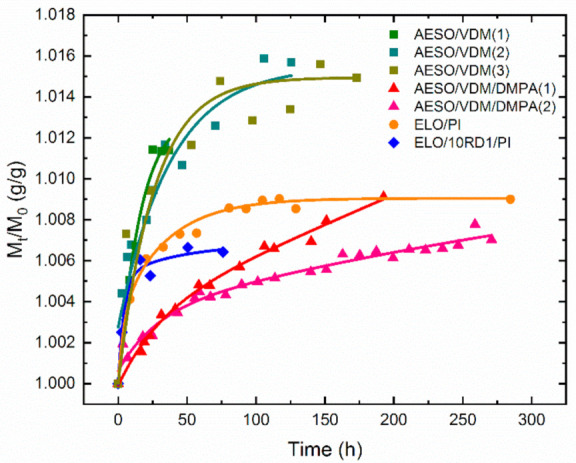
Time dependence of reduced weight *M*(*t*)/*M*(0) at swelling experiment for the investigated samples. AESO/VDM squares, AESO/VDM/DMPA triangles, ELO/PI dots, and ELO/10RD1/PI diamonds.

Swelling studies were performed several times to find out additional information regarding the stability of the sample structure. The results of the swelling experiments are shown in Figure 9 and in Table 2. The *S* parameters, commonly used to characterize the swelling ability of a polymer in a given solvent, are gathered in Table 2. *S* is defined as *S* = 100*(M_swollen_ − M_dry_)/M_dry_* (in %), where *M_swollen_* is the maximal weight of the sample saturated in solvent and *M_dry_* is its weight after drying. For desorption processes in PALS measurements, the initial (maximal) weight was used as *M_swollen_* and minimal weight, after full desorption in vacuum before the last PALS measurement, as a *M_dry_*. The *S* parameter in Table 2 confirmed that the greatest sorption capacity belongs to the AESO/VDM polymer matrix.

Compared to other samples, the AESO/VDM/DMPA showed an increasing trend over the entire measured time interval. In the case of later repeated swelling after about 50 h of sorption, this sample showed a lower weight gain compared to the primary swelling. Sample AESO/VDM, which is similar in composition but synthesized without the photoinitiator DMPA, clearly shows a higher water sorption capacity, which can be explained by the less cross-linking efficiency. However, we found that the structure of the AESO/VDM network was much more stable since the parameters remained constant even after the third swelling cycle. In the sample AESO/VDM/DMPA, which has a higher cross-linking density, it can be stated that the network bonds are less stable. This explains the unsaturated course of the dependence *M(t)* and also the decreased values of *M(t)* in the second swelling cycle. 

Both samples ELO/PI and ELO/10RD1/PI contained a photoinitiator (PI). ELO/10RD1/PI contained trimethylolpropane triglycidyl ether (RD1) which increased the degree of cross-linking. This was verified by the lower saturated weight level compared to ELO/PI. The differences between these two samples in terms of saturated mass value were smaller than in the case of samples AESO/VDM and AESO/VDM/DMPA.

The desorption of water in the samples was studied using two methodologies. First, the weight of the sample was recorded over time in the air at ambient laboratory conditions (atmospheric pressure, 23 °C, 60% relative humidity) for the comparison of sorption and desorption processes and their influence on the diffusion constant (AESO/VDM (3), Table 3).

Second, after the full adsorption of a sample (final swelling stage), PALS measurements were performed. The next partial water desorption was made in a low-vacuum chamber (approx. 0.1 atm). Then the sample was weighed for determination of water content and measured by PALS. Before and after each PALS measurement, the weight of the sample was recorded to test the conditions required for stability. After that, the next partial desorption was performed under low vacuum. These cycles were performed until weight reached a constant minimum. The last desorption was done in vacuum (0.01 atm, 1 h). PALS measurements were performed in a hermetically closed chamber in air.

**Figure 10:**
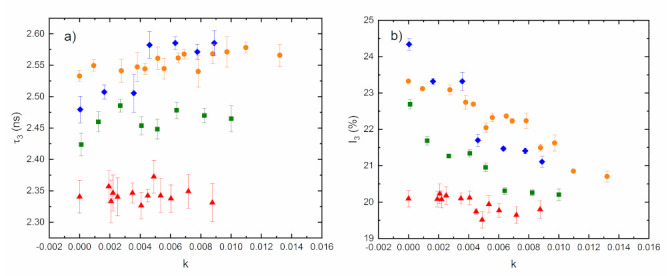
The o-Ps lifetime (**a**) and their relative intensity *I_3_* (**b**) as a function of water content expressed by *k* for the investigated samples. AESO/VDM squares, AESO/VDM/DMPA triangles, ELO/PI dots, and ELO/10RD1/PI diamonds.

The annihilation characteristics of individual samples depending on the amount of water (expressed by filling coefficient *k*) were obtained. The filling coefficient is defined here as: *k* = *M_water_/(M_water_ + M_drymatrix_)*, where *M_water_* is the mass of water inside of the sample and *M_drymatrix_* is the mass of the polymer sample with minimal value. The results for all samples for both the o-Ps lifetime and their relative intensity are shown in Figure 10.

**Figure 11:**
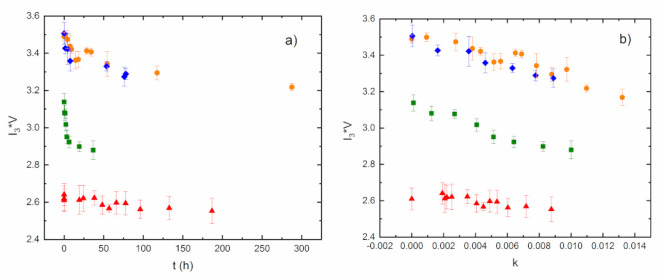
The reduced free volume fraction *fv*/*C* for the investigated samples as a function of time at swelling (reconstructed) (**a**) and as a function of water content expressed by *k* (**b**) AESO/VDM squares, AESO/VDM/DMPA triangles, ELO/PI dots, and ELO/10RD1/PI diamonds.

Based on the time-dependent change of the normalized mass *M(t)/M(0)* (curves in Figure 9, swelling of the samples), the time dependence of the PALS parameters during the water sorption into the material was constructed on the assumption that the annihilation parameters in the sample are the same for the given amounts of water in the desorption (measured PALS values) as well as in the swelling (reconstructed PALS values) processes. Figure 11 depicts the reduction of the free volume fraction *fv/C* for all the investigated samples with time. It is evident that the free volume fraction decreases with increasing water content as the voids are gradually filled with water molecules, with the exception of AESO/VDM/DMPA, where the fraction decrease is negligible.

The diffusion constant for water in the investigated samples was estimated according to the formula:

(5) $$D=d^{2}/12\tau ,$$

where *D* is the diffusion constant, *d* is the thickness of the sample (3 mm), and *τ* is the mean first passage time. The last one was determined by numerical integration of dependence *P(t) = [M(t)–M(∞)]/[M(0)–M(∞)]*, where *M(0)* and *M(∞)* represent the wet and dry weights of the sample, respectively. *M(t)* is the mass of the sample at time *t*. A detailed explanation of the concepts and the derivation of the formula for calculating *D* is shown in Appendix S1 (Supplementary material). The results for all samples are shown in Table 3.

The results for AESO/VDM sample show that the processes of water penetration into the sample (swelling) and water leakage (desorption in air) give a very similar value of the constant D. ELO/10RD1/PI has the largest coefficient D despite that this material has a low sorption capacity (low *S* and *M(t)_max_/M(0)*).

### 3.5. Investigation of the Polymers as Immobilization Matrixes in Enzymatic Biosensors Construction

A commercial laccase (EC 1.10.3.2) from *Trametes versicolor* was chosen as a model catalytic element to investigate the impact of polymer structure on the efficacy of enzyme immobilization in the construction of the amperometric biosensors. This well-known enzyme is widely used in biosensorics due to its ability to oxidize a variety of organic substrates in the presence of molecular oxygen that is reduced to water without the requirement of exogenous cofactors or electron transfer mediators. The enzymatic bioelectrodes were prepared according to the procedure described in the experimental section. However, it is important to mention that AESO/VDM/DMPA polymer could not be employed as an immobilization matrix due to its physical properties. The polymer has a characteristic rigid plastic-like structure that does not allow control of the material thickness.

**Figure 12:**
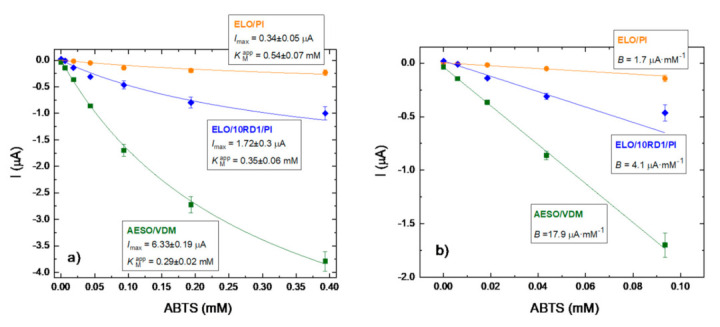
Calibration curves of chronoamperometric response (**a**) and analysis of linearity range and sensitivity (**b**) on the increasing concentrations of ABTS for the laccase bioelectrodes formed with different polymer matrixes. Conditions: working potential −100 mV vs. Ag/AgCl (3 M KCl), 50 mM acetate buffer, pH 4.5 at 23 °C and constant stirring.

For the chronoamperometric characterization of the constructed laccase-based bioelectrodes, a working potential of −100 mV vs. Ag/AgCl (3M KCl) was selected as the optimal one based on our previous experience in the evaluation of mediatorless ELO/laccase-based biosensors [15]. The bioelectrodes were characterized by the main sensor operational parameters: a maximal current at substrate saturation (*I_max_*); apparent Michaelis–Menten constant (*K_M_^app^*); sensitivity; and linearity. The typical response of the bioelectrodes based on different polymer matrixes toward ABTS as a specific laccase substrate is presented in Figure 12.

The calibration curves shown in Figure 12 correspond to the typical chronoamperogramms (Figure S11, supplementary material) of the bioelectrodes constructed by means of ELO/PI, ELO/10RD1/PI, as well as AESO/VDM. Both of them have demonstrated a significant difference in their operational properties. The bioelectrodes based on AESO/VDM have shown a 3.7-fold higher *I*_max_ value compared to the one based on ELO/10RD1/PI and more than 18-fold higher than ELO/PI-modified bioelectrodes (6.33 µA vs. 1.72 µA and 0.34 µA, respectively). The listed electrodes were characterized by the *K_M_^app^* values: 0.29 mM; 0.35 mM; 0.53 mM of ABTS, respectively. Interestingly, *K_M_^app^* value for AESO/VDM matrix is the same when compared with *K*_M_ of ABTS substrate for laccase from *T. versicolor* in solution (0.29 mM) [53]. Such a high affinity of the constructed bioelectrodes indicates very comfortable conditions for the immobilized enzyme catalysis in the polymeric matrix. The sensitivity (recalculated as a relation of calibration slope *B* to working electrode area 7.3 mm^2^) is the most important biosensor characteristic and was found to be: 2452 A⋅M^−1^⋅m^−2^ for AESO/VDM, 562 A⋅M^−1^⋅m^−2^ for ELO/10RD1/PI, and 233 A⋅M^−1^⋅m^−2^ for ELO/PI polymer matrix (Figure 12b and Table 4). Hence, bioelectrodes based on AESO/VDM matrix possessed 4.4-fold higher sensitivity when compared to ELO/10RD1/PI and one order higher compared to ELO/PI matrixes, respectively. The linear range of the bioelectrodes was found in the frames of 10 µM to 10 mM of ABTS (Figure 12b, Table 4).

The wide linear frames and very high sensitivity of the bioelectrodes based on AESO/VDM make them very promising in the analysis of toxic phenol derivatives in real samples of drinking water or wastewater.

## 4. Conclusions

In this work, the transitions to the glassy state and the adsorption properties of the vegetable oil-based samples using positron annihilation were investigated. The investigation of these processes is part of a wider comprehensive study of the properties of vegetable oil-based samples selected as suitable matrixes candidates for biosensors [2]. The following conclusions can be depicted from our results.

In terms of stability, the MELT analysis of spectra for virgin and thermal treated samples suggests high resistance of the biopolymer matrix to changing thermal conditions (toward low temperatures). Temperature-dependent tests allowed the determination of phase transition temperatures and the determination of quantitative parameters of local free volumes in polymers. The *T_g_* temperature in the investigated samples was found to be very similar. It should be noted that the AESO/VDM polymer had the largest free volumes. At room temperature, the size of free volume in AESO/VDM is 180 Å^3^, while for two ELO-based polymers, it is approximately 165 Å^3^. The last polymer, AESO/VDM/DMPA, clearly has the lowest free volumes, 140 Å^3^. If the size of free volumes of polymer matrix plays a role in obtaining the best biosensor detection parameters [15,16,17,18], the AESO/VDM seems to be the perfect candidate for their production.

The influence of humidity on the polymers has been analyzed. In three samples subjected to the process of removing water molecules by degassing, the o-Ps intensity increases significantly up to exceeding the reference level. Continuation of the degassing process leads to a further increase in o-Ps intensity, and the expected saturation level is higher than the reference level. Water molecules are removed from the AESO/VDM sample much faster in comparison with the other samples, after just the first h, the *I*_3_ intensity reaches the reference sample level. This effect does not occur in the AESO/VDM/DMPA sample. Volume sizes remain unchanged in ELO/PI, ELO/10RD1/PI, and AESO/VDM/DMPA samples. The presence of water permanently modifies (reduces) free volumes only in the AESO/VDM matrix. The nanostructure of this sample was found to be the most sensitive to water.

The swelling in water along with PALS parameters measurements during stepwise desorption of water from investigated materials made possible the estimation of water diffusion coefficients *D*. Additionally, the changes in free volume at different water content at 23 °C and normal atmospheric pressure were analyzed. The swelling has shown that as the amount of water in the samples increases, local free volumes become clogged (their share of the total free volume decreases, e.g., the *I*_3_ or *fv*/*C* decreases), and the polymer chains expand around them. This conclusion was made based on the increase of local free volume sizes where water did not get (not clogged holes). The only exception is AESO/VDM/DMPA, which did not show appreciable change.

Based on the results of testing the polymers as immobilization matrixes in enzymatic biosensors’ construction, it can be concluded that the best material for this is AESO/VDM, which is characterized by the large size of free volumes and high-water content. That makes sense as the presence of water molecules is extremely important to support the native (three-dimension) enzyme structure due to noncovalent (hydrophilic/hydrophobic) interactions of the protein’s amino acid residues with polar molecules of H_2_O. This also supports some enzyme flexibility for its optimal catalytic and electron-transfer ability. Thus, the AESO/VDM-based bioelectrodes were characterized by significant improvements in the main sensor characteristics in comparison with the ELO/PI or ELO/10RD1/PI. On the other hand, the polymer AESO/VDM/DMPA (with opposed characteristics vs. AESO/VDM) is completely unsuitable for usage in biosensors construction. PALS analysis predicted very accurately which vegetable oil-based polymer could provide a better immobilization matrix. This was confirmed by the experimental construction of vegetable oil-based amperometric biosensors.

## Figures and Tables

**Table 1:** The Parameters of the Exponential Curve Given by Equation 4 Fitted into the Experimental Data Presented in Figure 7 and Figure 8.

Sample	Process	*Θ*, h
ELO/PI	sorption	39.0
	desorption	40.4
ELO/10RD1/PI	sorption	79.9
	desorption	100.7
AESO/VDM	sorption	28.1
	desorption	20.8

**Table 2:** The Swelling Characteristics of the Investigated Polymer Samples. Numbers in Brackets in Some Cases Mean the Order of Repeated Swelling.

Sample	*M*(*t*)_max_/*M*(0) (g/g), swell.	*S*, %
AESO/VDM (2)	1.0150	1.432
AESO/VDM/DMPA (1)	1.0091	0.969
ELO/PI	1.0090	1.340
ELO/10RD1/PI	1.0065	0.911

**Table 3:** The Estimated Diffusion Constant *D* and Mean First Passage Time *τ* for Water in the Investigated Polymer Samples at Room Temperature (23 °C).

Sample	*τ*, h	*D*, m^2^·s^−1^	Process
ELO/PI	22.55	9.24 × 10^−12^	swelling
ELO/10RD1/PI	8.96	2.33 × 10^−11^	swelling
AESO/VDM (3)	28.3	7.36 × 10^−12^	swelling
AESO/VDM (3)	27.5	7.58 × 10^−12^	desorption in air
AESO/VDM/DMPA	40.4	5.16 × 10^−12^	swelling

**Table 4:** Comparison of the Main Operational Parameters of the Biosensors Based on Different Polymer Matrixes.

Polymer Matrix	*I*_max_, μA	*K*_M_^app^, mM	Slope (*B*), μA·mM^−1^	Sensitivity, A·M^−1^·m^−2^	Linearity, mM
ELO/PI	0.34 ± 0.05	0.54 ± 0.07	1.7	233	0.02–0.10
ELO/10RD1/PI	1.72 ± 0.3	0.35 ± 0.06	4.1	562	0.006–0.10
AESO/VDM	6.33 ± 0.19	0.29 ± 0.02	17.9	2452	0.006–0.10
AESO/VDM/DMPA	–	–	–	–	–

## Data Availability

The data presented in this study are available on request from the corresponding authors.

## Supplementary Materials

The following supporting information can be downloaded at: https://www.mdpi.com/article/10.3390/ma15196607/s1, Figure S1: Photograph of the four tested samples; Figure S2: Preparation of samples for measurements.; Figure S3: Test tube containing solidified deionized water. Step of degassing vapors from the sample to remove O_2_ molecules from water.; Figure S4: The o-Ps lifetime and intensity as a function of time for four investigated samples at −150 °C.; Figure S5: ELO/PI: the o-Ps lifetime and intensity as a function of increasing and decreasing temperature.; Figure S6: ELO/10RD1/PI: the o-Ps lifetime and intensity as a function of increasing and decreasing temperature.; Figure S7: Upper part of figure: the o-Ps lifetime as a function of increasing and decreasing temperature. Bottom part of the figure: DSC thermogram of ELO/PI and ELO/10RD1/PI consisting of cooling scan followed by heating one with cooling/heating rates 10 K/min.; Figure S8: AESO/VDM: the o-Ps lifetime and intensity as a function of increasing and decreasing temperature.; Figure S9: AESO/VDM/DMPA: the o-Ps lifetime and intensity as a function of increasing and decreasing temperature.; Figure S10: Dependence of lifetimes and o-Ps intensity as a function of increasing temperature.; Figure S11: Typical chronoamperometric response of the bioelectrodes formed with different polymer matrices on the increasing concentrations of ABTS. Conditions: working potential −100 mV vs. Ag/AgCl (3M KCl), 50 mM acetate buffer, pH 4.5 at 23 °C, at constant stirring. Table S1: The glass transition temperature *T*_g_ was determined on the basis of changes in o-Ps parameters; Appendix S1: The method calculating the diffusion constant *D*.
