# Supplementary material for: UV-Cured Green Polymers for Biosensorics: Correlation of Operational Parameters of Highly Sensitive Biosensors with Nano-Volumes and Adsorption Properties
Source: Materials (Basel). 2022 Sep 23;15(19):6607. doi: 10.3390/ma15196607 (PMC9572821; doi:10.3390/ma15196607)
Supplement: Supplementary file 1 [file materials-15-06607-s001.zip › materials-1917304-supplementary.pdf]

materials

MDPI

Supplementary Materials

UV-Cured Green Polymers for Biosensorics: Correlation of Operational Parameters of Highly Sensitive Biosensors with Nano-Volumes and Adsorption Properties

Magdalena Goździuk [1], Taras Kavetskyy [2,3,*], Daniel Massana Roquero [4], Oleh Smutok [4,5], Mykhailo Gonchar [2,5], David P. Královič [6], Helena Švajdlenková [7], Ondrej Šauša [6,8], Pavol Kalinay [8], Hamed Nosrati [9], Migle Lebedevaite [10], Sigita Grauzeliene [10], Jolita Ostrauskaite [10], Arnold Kiv [11] and Bożena Zgardzińska [1,*]

1 Institute of Physics, Maria Curie-Sklodowska University, 20-031 Lublin, Poland
2 Department of Biology and Chemistry, Drohobych Ivan Franko State Pedagogical University, 82100 Drohobych, Ukraine
3 Department of Materials Engineering, The John Paul II Catholic University of Lublin, 20-950 Lublin, Poland
4 Department of Chemistry and Biomolecular Science, Clarkson University, Potsdam, NY 13699-5810, USA
5 Department of Analytical Biotechnology, Institute of Cell Biology, National Academy of Sciences of Ukraine, 79005 Lviv, Ukraine
6 Department of Nuclear Chemistry, Comenius University in Bratislava, 84215 Bratislava, Slovakia
7 Polymer Institute, Slovak Academy of Sciences, 84541 Bratislava, Slovakia
8 Institute of Physics, Slovak Academy of Sciences, 84511 Bratislava, Slovakia
9 Department of Pharmaceutical Biomaterials, School of Pharmacy, Zanjan University of Medical Sciences, 45139-56111 Zanjan, Iran
10 Department of Polymer Chemistry and Technology, Kaunas University of Technology, 50254 Kaunas, Lithuania
11 Department of Innovation Technologies, South-Ukrainian K.D. Ushynsky National Pedagogical University, 65020 Odesa, Ukraine
* Correspondence: kavetskyy@yahoo.com (T.K.); bozena.zgardzinska@mail.umcs.pl (B.Z.); Tel.: +38-03244-37700 (T.K.); +48-81-537-6286 (B.Z.)

Citation: Goździuk, M.; Kavetskyy, T.; Roquero, D.M.; Smutok, O.; Gonchar, M.; Královič, D.P.; Švajdlenková, H.; Šauša, O.; Kalinay, P.; Nosrati, H.; et al. UV-Cured Green Polymers for Biosensorics: Correlation of Operational Parameters of Highly Sensitive Biosensors with Nano-Volumes and Adsorption Properties. *Materials* **2022**, *15*, 6607. https://doi.org/10.3390/ma15196607

Academic Editor: Béla Iván

Received: 30 August 2022
Accepted: 16 September 2022
Published: 23 September 2022

Publisher's Note: MDPI stays neutral with regard to jurisdictional claims in published maps and institutional affiliations.

Copyright: © 2022 by the authors. Licensee MDPI, Basel, Switzerland. This article is an open access article distributed under the terms and conditions of the Creative Commons Attribution (CC BY) license (https://creativecommons.org/licenses/by/4.0/).

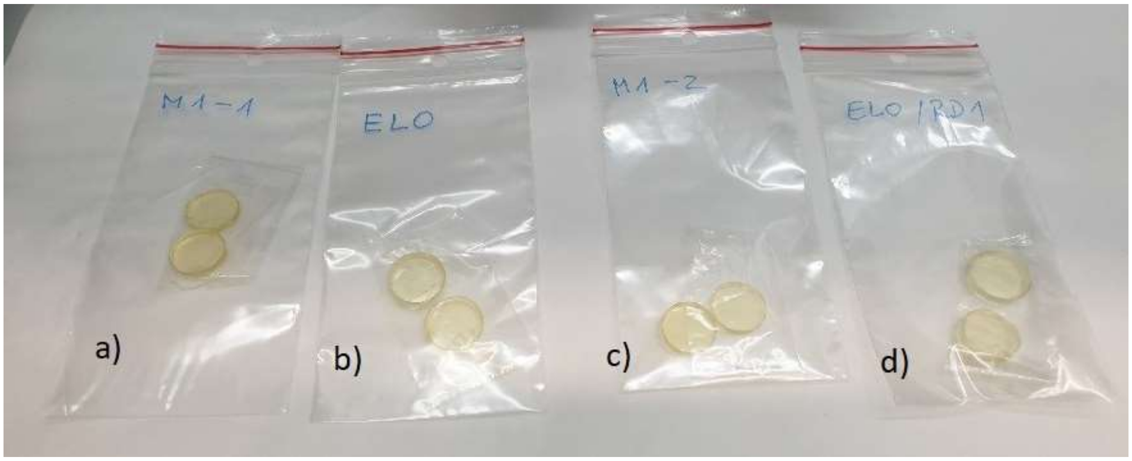

Figure S1. Photograph of the four tested samples: (**a**) AESO/VDM, (**b**) ELO/PI, (**c**) AESO/VDM/DMPA, (**d**) ELO/10RD1/PI.

a)
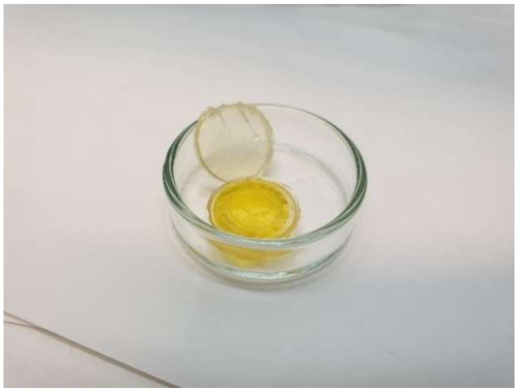

b)
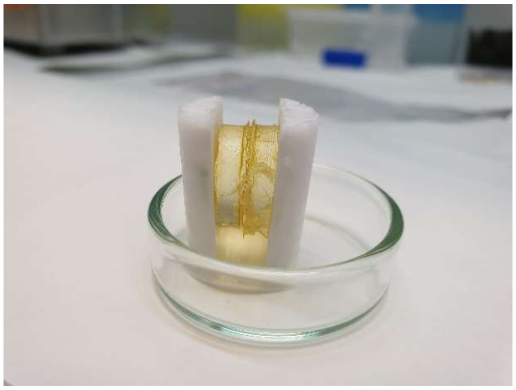

c)
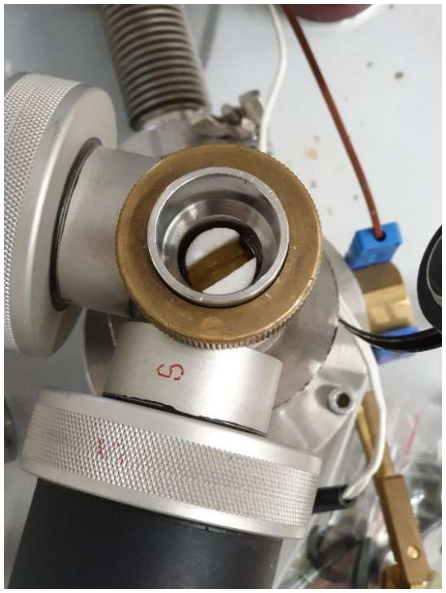

Figure S2. Preparation of samples for measurements: (**a**) sample with source, (**b**) sandwich geometry with Teflon sample stabilizers, and (**c**) sample in the chamber with visible scintillators.

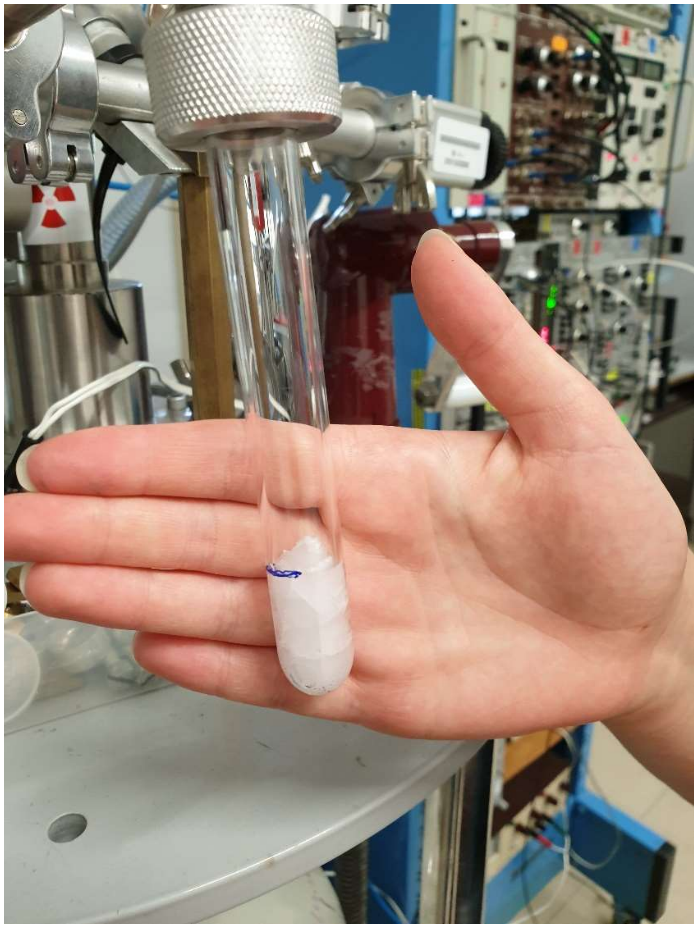

Figure S3. Test tube containing solidified deionized water. Step of degassing vapors from the sample to remove O_2 molecules from water.

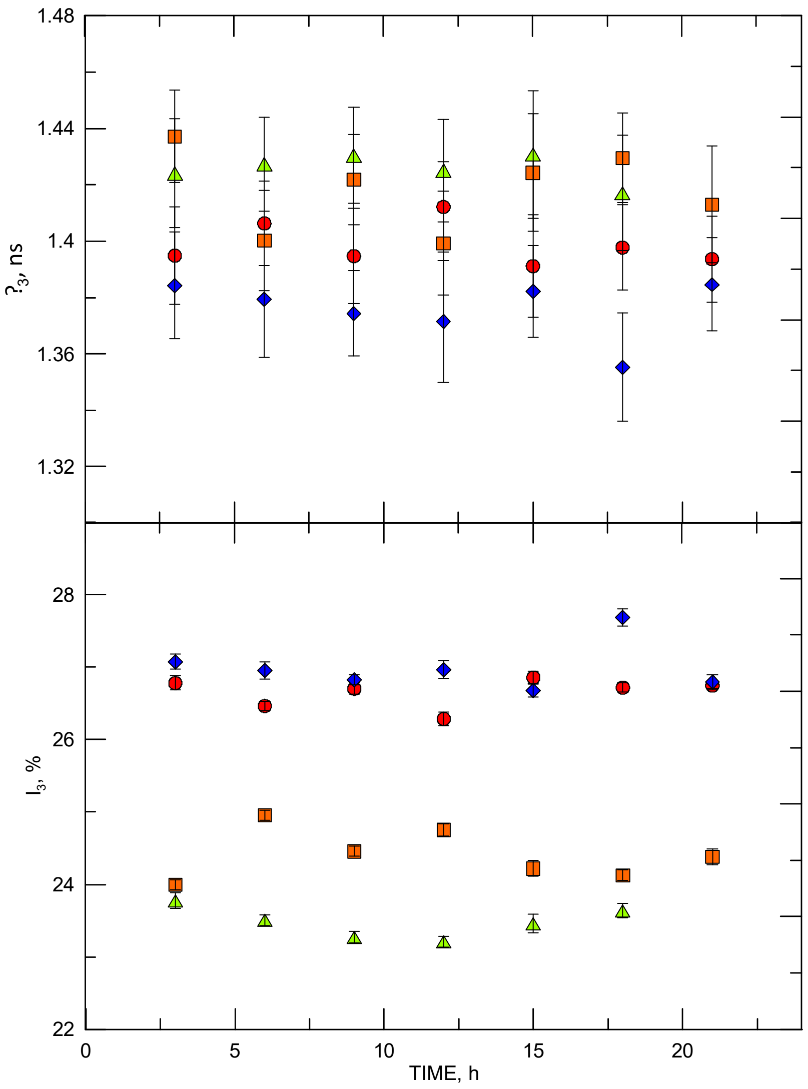

Figure S4. The o-Ps lifetime and intensity as a function of time for four investigated samples at -150 °C; dots – ELO/PI, diamonds – ELO/10RD1/PI, squares – AESO/VDM, and triangles – AESO/VDM/DMPA.

Figure S4 shows time-changes of o-Ps lifetime and intensity in all tested samples at -150 °C. All samples during the measurement (over 20 h) did not show clear variability of the PALS parameters. The spectra presented here were summarized and analyzed by the MELT program.

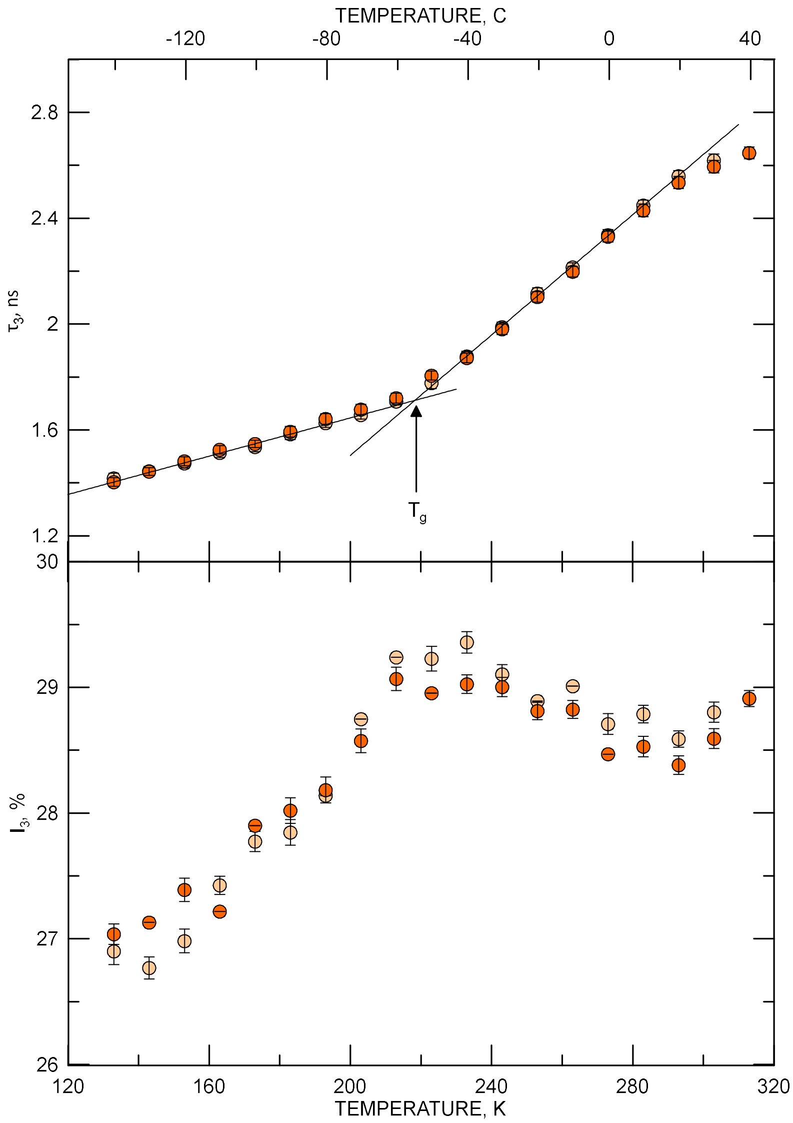

Figure S5. ELO/PI: the o-Ps lifetime and intensity as a function of increasing (orange dots) and decreasing (creamy dots) temperature.

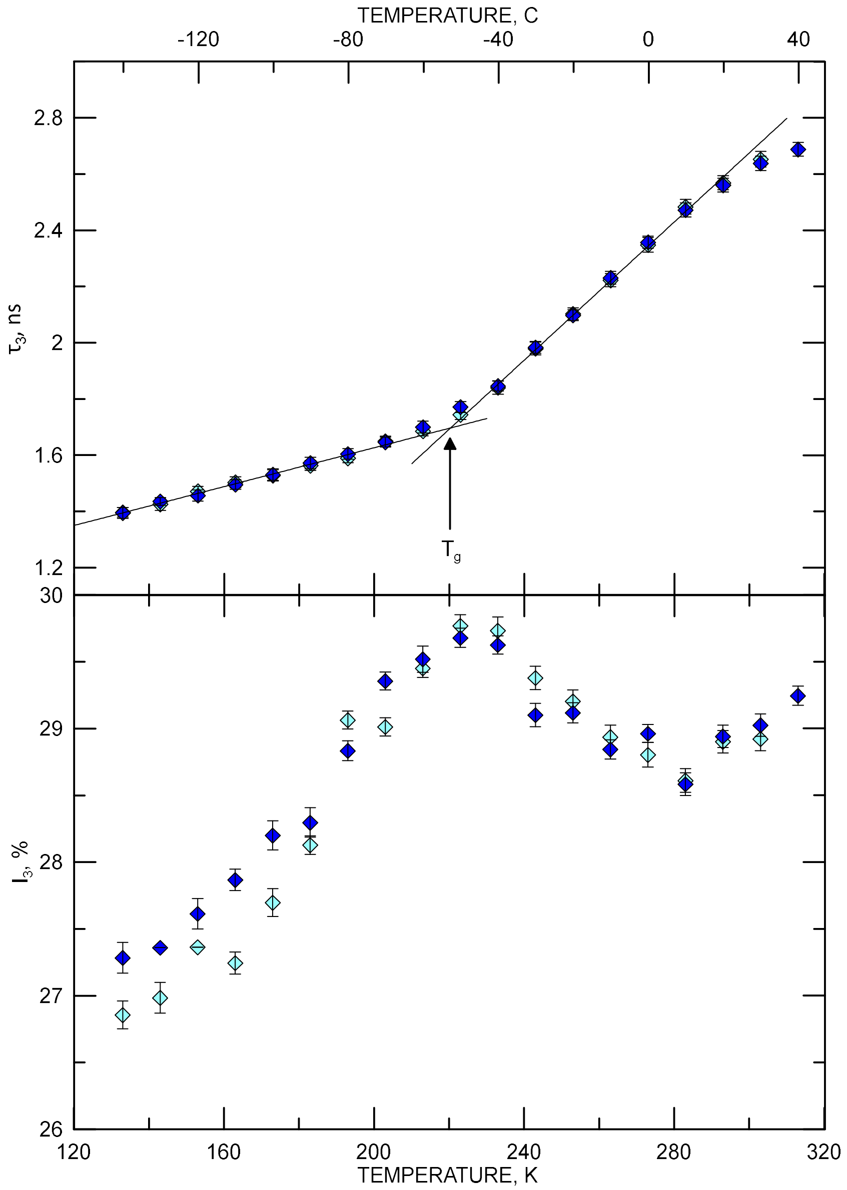

Figure S6. ELO/10RD1/PI: the o-Ps lifetime and intensity as a function of increasing (dark blue diamonds) and decreasing (light blue diamonds) temperature.

Table S1. The glass transition temperature T_g determined on the basis of changes in o-Ps parameters.

	ELO/PI	ELO/10RD1/PI	AESO/VDM	AESO/VDM/DMPA
**Increasing temperature cycle**	221.8 K	220.1 K	225.2 K	218.3 K
**Decreasing temperature cycle**	218.3 K	220.3 K	220.6 K	224.5 K

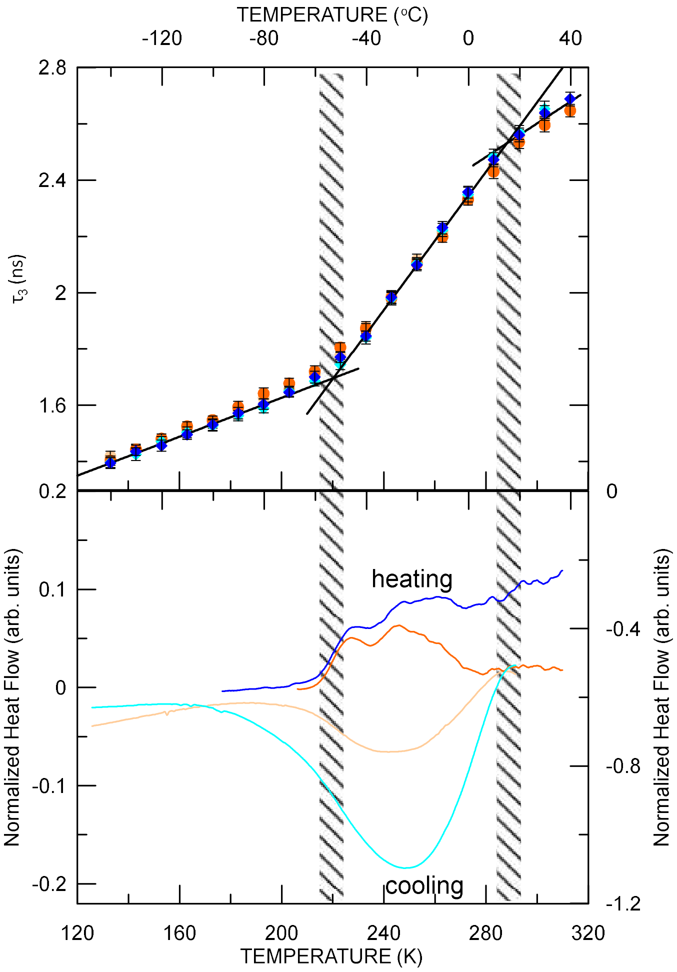

Figure S7. Upper part of figure: the o-Ps lifetime as a function of increasing (orange dots – ELO/PI; dark blue diamonds – ELO/10RD1/PI) and decreasing (creamy dots – ELO/PI; light blue diamonds – ELO/10RD1/PI) temperature. Bottom part of the figure: DSC thermogram of ELO/PI (blue lines) and ELO/10RD1/PI (orange lines) consisting of cooling scan followed by heating one with cooling/heating rates 10 K/min.

The calorimetric studies were performed using Differential Scanning Calorimetry DSC PYRIS Diamond Perkin Elmer, USA. The samples were sealed in aluminum pans. During the measurements an inert atmosphere surrounding the samples was maintained using He flow. To avoid the white frost of external elements of the system, the purging gas (N_2) was used. The studies of phase transitions were performed in the range +40 °C ÷ -150 °C with temperature changing rate of 10 K/min. The DSC results do not indicate the presence of two distinct phase transitions, but rather suggest the existence of one phase transition spanning approximately 70 K.

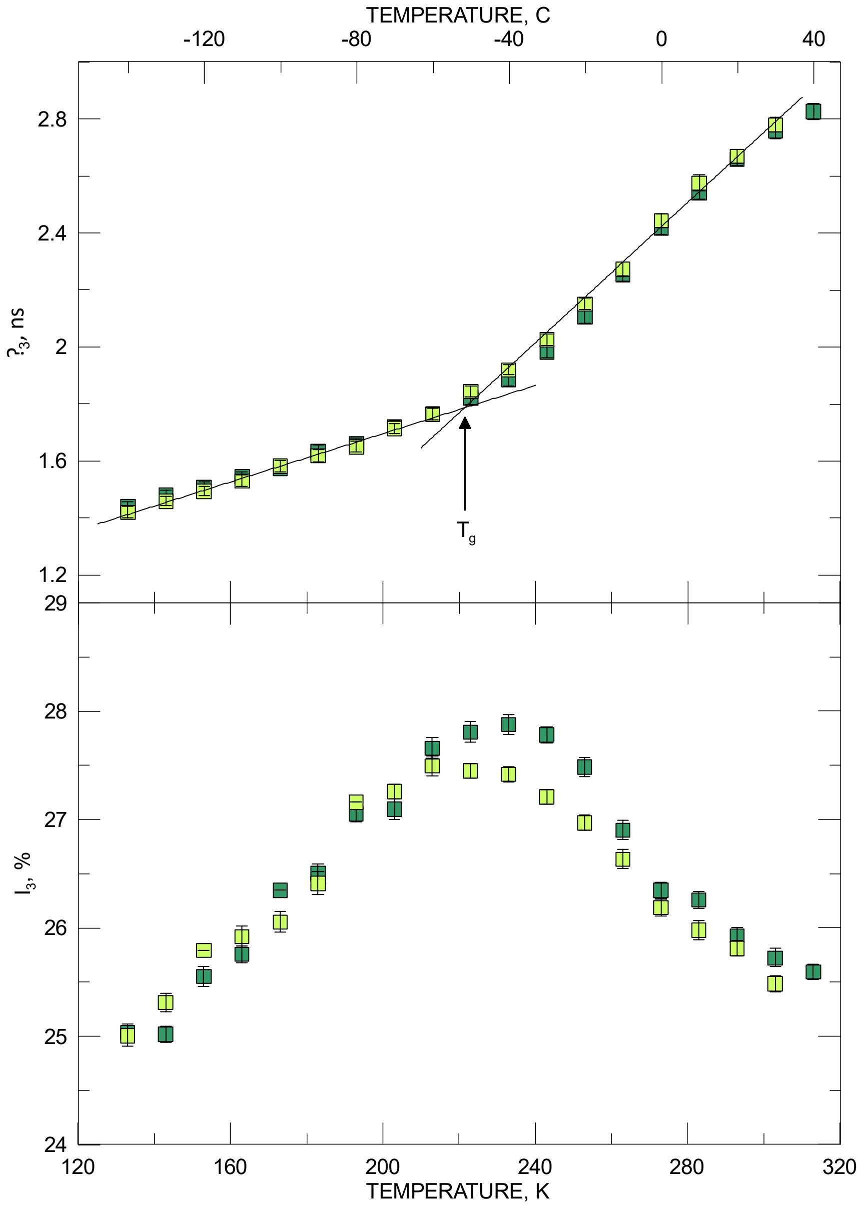

Figure S8. AESO/VDM: the o-Ps lifetime and intensity as a function of increasing (dark green squares) and decreasing (light green squares) temperature.

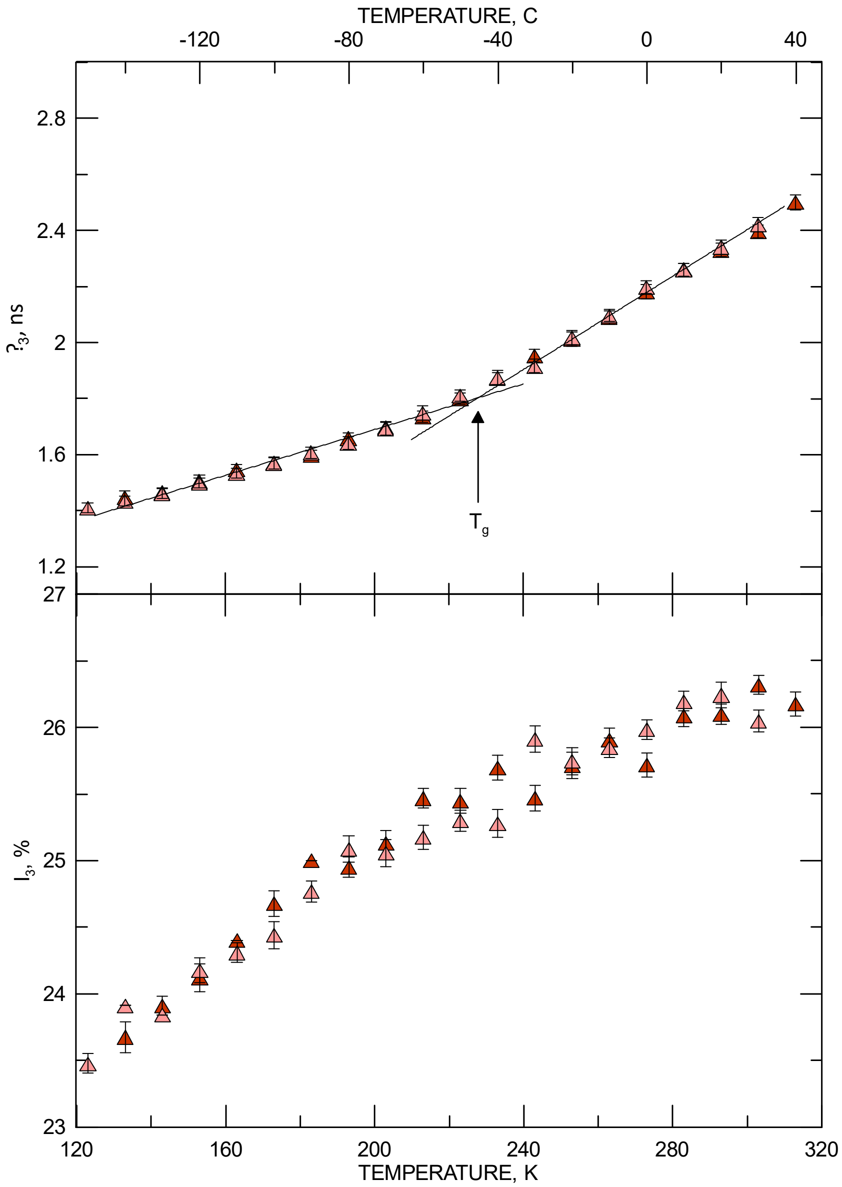

Figure S9. AESO/VDM/DMPA: the o-Ps lifetime and intensity as a function of increasing (red triangles) and decreasing (pink triangles) temperature.

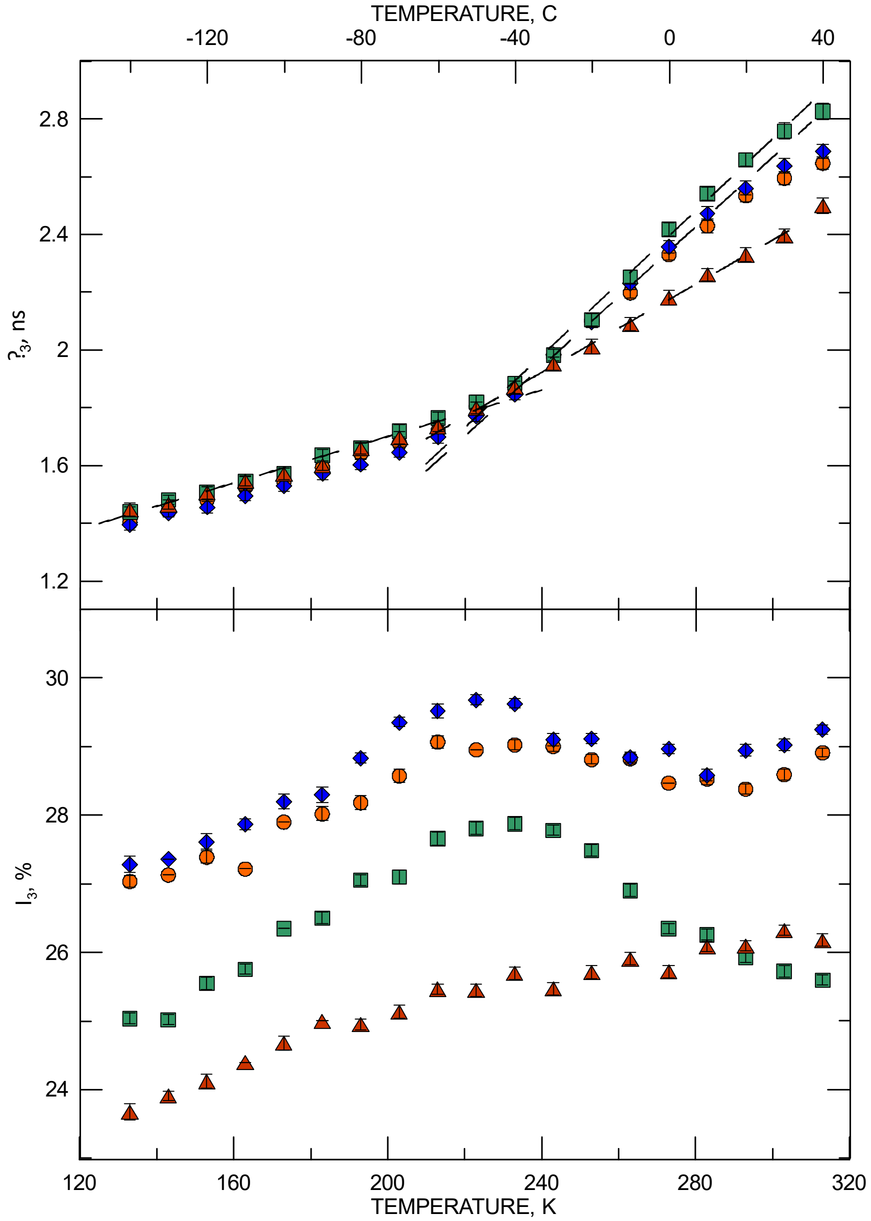

Figure S10. Dependence of lifetimes and o-Ps intensity as a function of increasing temperature (dots – ELO/PI, diamonds – ELO/10RD1/PI, squares – AESO/VDM, and triangles – AESO/VDM/DMPA).

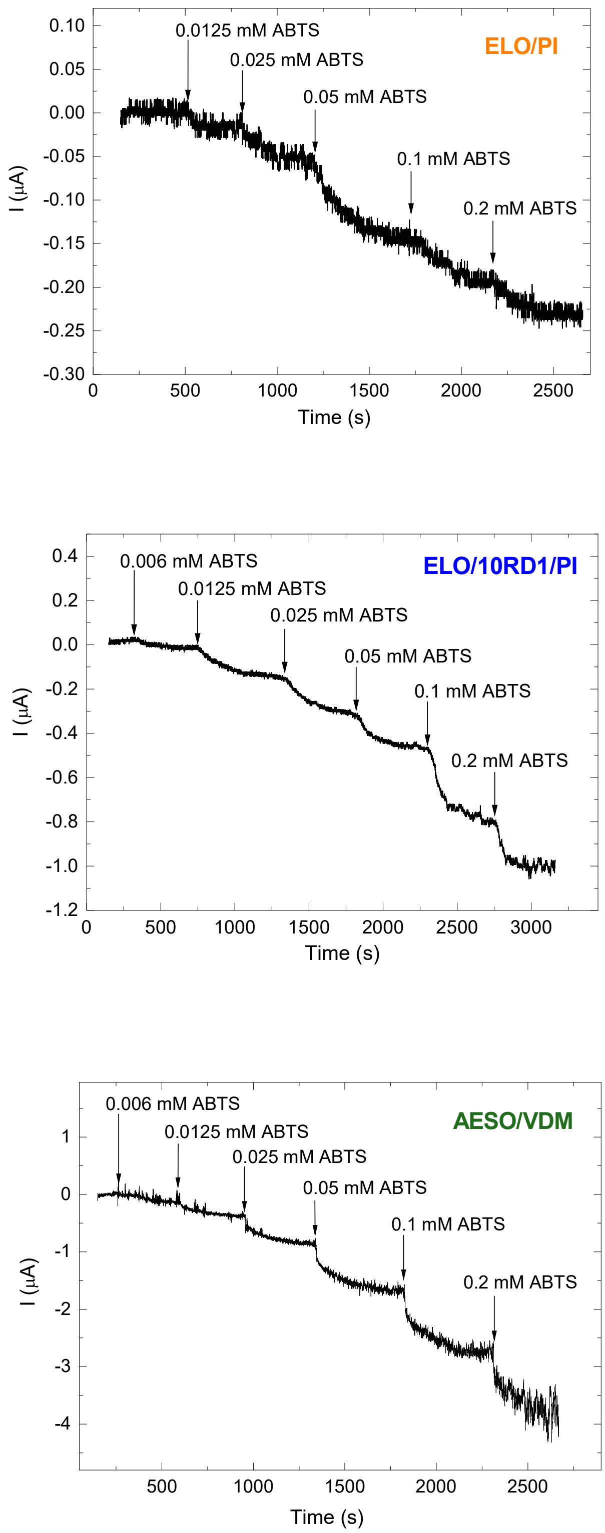

Figure S11. Typical chronoamperometric response of the bioelectrodes formed with different polymer matrices on the increasing concentrations of ABTS. Conditions: working potential -100 mV vs. Ag/AgCl (3M KCl), 50 mM acetate buffer, pH 4.5 at 23 °C, at constant stirring.

Appendix S1

The method calculating the diffusion constant D.

The time dependent function of the measured sample mass $M(t)$ is used for evaluation of the diffusion constant D of the water molecules absorbed in the sample applying the concept of the mean first passage time (MFPT) τ.

We first consider the process of **desorption**; a particle is present inside the sample at time $t=0$ and then it diffuses until hitting the boundary. At the moment of the first hit, it disappears from the sample (it immediately evaporates from the sample surface). This process is described by the survival probability $P(t)$, the probability that the particle is still (survives) in the sample at time t. Then the MFPT is defined as

$$\tau = \int_0^{\infty} t\left(-\frac{dP(t)}{dt}\right)dt = \int_0^{\infty} P(t)dt \tag{1}$$

integrating by parts. We suppose $P(0) = 1$ (particle is surely in the system), $P(t \to \infty) = 0$, and $-dP(t)/dt$ represents the probability density that the particle leaves the sample at time t.

In the one-particle approximation, we consider the water molecules not restricting one another. Then the sample mass $M(t)$ depends linearly on the survival probability $P(t)$; hence we get $P(t) = [\mathrm{M}(t) - M(\infty)]/[M(0) - M(\infty)]$; $M(0)$ and $M(\infty)$ represent the wet and dry weights of the sample, respectively. The numerical integration of the obtained $P(t)$ results in τ.

Considering diffusion of the molecules from depth of the sample to its surface to be the only process controlling decreasing its weight, the MFPT is calculated using the local function $T(\boldsymbol{r})$, the MFPT of a particle starting from the specific position $\boldsymbol{r}$ inside the sample. It is determined [1] as the solution of the equation

$$D \cdot \Delta \cdot T(\boldsymbol{r}) = -1 \tag{2}$$

with the Dirichlet boundary condition $T(\boldsymbol{r}) = 0$ at the sample surface, as the particle put at the surface is immediately leaving the system. Supposing the uniform initial distribution of the particle in the sample, the MFPT τ is $T(\boldsymbol{r})$ averaged over the volume Ω,

$$\tau = \frac{1}{|\Omega|} = \int_{\Omega} T(\boldsymbol{r})d\boldsymbol{r} \tag{3}$$

For the sample shaped as a thin cylinder of the width d and the radius $R >> d$, most particles diffuses towards the nearest surface in the axial direction, and we can adopt the one-dimensional picture in the axial coordinate $0<x<d$. The equation for $T(\boldsymbol{r})$ reduces to $Dd^2T(x)/dx^2 = -1$, which is easily solvable: $T(x) = x(d-x)/2D$ for the boundary conditions $T(0) = T(d) = 0$ at the sample surface. Averaging over the width d gives $\tau = d^2/12D$ and results in the final formula

$$D = \frac{d^2}{12\tau}. \tag{4}$$

The **absorption** of the water can be described as a similar process, where the vacancies diffuse out of the sample. Their diffusivity is the same as for the water molecules in the one-particle approximation, so one can use the same formulas for $P(t)$ and τ; of course, $M(0)$ represents now the initial weight of the dry sample and $M(\infty)>M(0)$ is the final weight of the wet sample.

Reference

1. Zwanzig, R. *Nonequilibrium Statistical Mechanics*; Oxford University Press: Oxford, UK, 2001.
